# Identification of N6-methylandenosine related lncRNA signatures for predicting the prognosis and therapy response in colorectal cancer patients

**DOI:** 10.3389/fgene.2022.947747

**Published:** 2022-09-30

**Authors:** Zhiyong Li, Yang Liu, Huijie Yi, Ting Cai, Yunwei Wei

**Affiliations:** ^1^ Department of Oncological and Endoscopic Surgery, The First Affiliated Hospital of Harbin Medical University, Harbin, China; ^2^ Pancreatic and Gastrointestinal Surgery Division, HwaMei Hospital, University of Chinese Academy of Science, Ningbo, China; ^3^ Translational Medicine Research and Cooperation Center of Northern China, Heilongjiang Academy of Medical Sciences, Harbin, China; ^4^ Peking University School of Nursing, Beijing, China; ^5^ Respiratory and Critical Care Medicine, Peking University People’s Hospital, Beijing, China; ^6^ Department of Experimental Medical Science, HwaMei Hospital,University of Chinese Academy of Sciences, Ningbo, China; ^7^ Key Laboratory of Diagnosis and Treatment of Digestive System Tumors, Ningbo, Zhejiang, China

**Keywords:** colorectal cancer1, N6-methylandenosine2, long non-coding RNA3, prognostic4, genetic alteration5, immune cell infiltration6, single-cell sequencing7, 5-fluorouracil8

## Abstract

Despite recent advances in surgical and multimodal therapies, the overall survival (OS) of advanced colorectal cancer (CRC) patients remains low. Thus, discerning sensitive prognostic biomarkers to give the optimistic treatment for CRC patients is extremely critical. N6-methyladenosine (m6A) and long noncoding RNAs (lncRNAs) play an important role in CRC progression. Nonetheless, few studies have focused on the impact of m6A-related lncRNAs on the prognosis, tumor microenvironment (TME) and treatment of CRC. In this study, 1707 m6A-related lncRNAs were identified through Pearson correlation analysis and Weighted co-expression network analysis (WGCNA) using The Cancer Genome Atlas (TCGA) cohort. Then, 28 m6A-related prognostic lncRNAs were screened by univariate Cox regression analysis, followed by identifying two clusters by consensus clustering analysis. A prognostic model consisted of 8 lncRNA signatures was constructed by the least absolute shrinkage and selection operator (LASSO). Kaplan–Meier curve analysis and a nomogram were performed to investigate the prognostic ability of this model. The risk score of prognostic model act as an independent risk factor for OS rate. Functional enrichment analysis indicated that lncRNA signatures related tumor immunity. The low-risk group characterized by increased microsatellite instability-high (MSI-H), mutation burden, and immunity activation, indicated favorable odds of OS. Moreover, the lncRNA signatures were significantly associated with the cancer stem cell (CSC) index and drug sensitivity. In addition, 3 common immune genes shared by the lncRNA signatures were screened out. We found that these immune genes were widely distributed in 2 cell types of TME. Finally, a ceRNA network was constructed to identify ZEB1-AS1 regulatory axis in CRC. We found that ZEB1-AS1 was significantly overexpressed in tumor tissues, and was related to the metastasis of EMT and the chemoresistance of 5-Fu in CRC. Therefore, our study demonstrated the important role of m6A-related lncRNAs in TME remodeling. Moreover, these results illustrated the levels of ZEB1-AS1 might be valuable for predicting the progression and prognosis of CRC, and further provided a new target for the diagnosis and treatment of CRC patients.

## Introduction

Colorectal cancer (CRC) is one of the most common malignant tumors and the second leading cause of cancer-related death worldwide (Siegel et al., 2013). The morbidity rate of CRC remains on the rise: currently, nearly 1.8 million people are diagnosed and more than 900,000 people die each year from CRC (Sung et al., 2021). Currently, the therapy methods for CRC include surgery, neoadjuvant chemoradiotherapy, postoperative chemoradiotherapy, targeted therapy, and immunotherapy (Tang et al., 2015). Despite recent advances in surgical and multimodal therapies, the 5-year survival rate remains poor for patients with postoperative recurrence and advanced CRC patients (Ganesh et al., 2019; Zhang et al., 2019). The TMN stage system has been widely used for clinical practice to predict patient prognosis and therapy decision-making. Dishearteningly, CRC has a complex status due to its heterogeneity; for instance, right-hemi and left-hemi CRC patients, even at the same stage, can show significantly different outcomes (Gallois et al., 2018). Thus, the development of sensitive biomarkers to accurately predict the prognosis and to monitor therapeutic effects in CRC patients is needed. Notably, one of the most important factors in the tumorigenesis and progression of CRC is driven by the accumulation of epigenetic changes, and epigenetic alterations can be developed as a clinical biomarker for prognostic and therapeutic uses (Jung et al., 2020).

N6-methyladenosine (m6A) serves as one of the most common and abundant epigenetic-methylation modifications, playing a crucial role in RNA processing, transport, and other functions (Zeng et al., 2018; Sun et al., 2019). m6A modification is regulated by methyltransferase (writers), demethylase (erasers), and binding proteins (readers) (Yang et al., 2018). Many studies have revealed that m6A modifications are associated with the development and prognosis of CRC (Li et al., 2019; Tsuruta et al., 2020). In addition, lncRNAs serve as a category of RNAs that do not encode proteins, and it has been reported that the aberrant expression of lncRNAs is related to the tumorigenesis, malignant progression, and poor prognosis of CRC patients (Bhan et al., 2017; Zhou et al., 2020). Indeed, the one important mechanism for achieving m6A functions in the cell involves modifying the lncRNAs(Zhou et al., 2016). Identification of specific lncRNA biomarkers associated with the prognosis of CRC would be of great clinical significance. Therefore, studies on m6A-related lncRNAs should be a focus if potential prognostic biomarkers for CRC patients are to be discovered. As is known, the tumor microenvironment (TME), which consists of stromal and immune cells, can affect the progression of cancer, and further provides a potential immunotherapy target (Nicholas et al., 2016). However, few studies have investigated the immune cell infiltration of m6A-related prognostic lncRNAs in CRC (Zhang et al., 2021a; Zeng et al., 2021), which might play an important role in tumorigenesis and malignant progression.

Here, we established clustering subtypes based on m6A-related lncRNAs to determine the relationships between the clustering subtypes, TME scores, prognosis, and immune cell infiltration, and further developed a CRC prognostic algorithm by using 8 m6A-related lncRNA signatures. This m6A-related lncRNAs algorithm showed considerable performance, which was confirmed by internal validation. In addition, the algorithm determined that high-risk patients were characterized by poor immune cell infiltration and clinical outcomes. Further, a nomogram including clinical risk factors together with an algorithm risk score was built, it was equipped with clinical utility and showed better performance. In addition. we explored the expression of 3 immune genes shared by the lncRNA signatures in different cell types of TME, which explained the mechanism of action of m6A-related lncRNAs. Finally, we reported that ZEB1-AS1 promoted carcinogenesis, metastasis of EMT and the chemoresistance of 5-Fu in CRC. Overall, the findings of this study suggest that the potential relationships between m6A-related lncRNAs, prognosis, and the TME of CRC patients, which may help developing novel therapeutic strategies for improving the overall clinical outcome of this disease.

## Materials and methods

### Data sources preparation and preprocessing

The RNA transcriptome dataset, including 480 tumor samples and 41 normal samples, as well as corresponding clinical information regarding CRC, were downloaded from the Cancer Genome Atlas (TCGA, https://portal.gdc.cancer.gov/) data portal. The other clinical characteristics, including the disease-free interval (DFI), progression-free interval (PFI), and disease-specific survival (DSS) of the 545 CRC patients, were downloaded from the USCS Xena website (https://xena.ucsc.edu/welcome-to-ucsc-xena). To distinguish between protein-coding genes and lncRNA genes for subsequent analyses, GTF files from Ensembl (https://asia.ensembl.org) were downloaded. CRC patients with missing survival values as well as a survival time of 0 days were excluded for these analyses. The data acquisition of the list of the immune genes was downloaded from the ImmPort database (https://www.immport.org). The 39 m6A regulator genes were identified from the published literature (Tu et al., 2020; Zhang et al., 2021b; Wang et al., 2021; Yu et al., 2021; Zhang and Yang, 2021). The m6A regulators contain 9 writers (METTL3, METTL14, METTL16, WTAP, VIRMA, ZC3H13, RBM15, RBM15B, and PCIF1), 27 readers (YTHDC1, YTHDC2, YTHDF1, YTHDF2, YTHDF3, HNRNPC, FMR1, LRPPRC, HNRNPA2B1, IGF2BP1, IGF2BP2, IGF2BP3, RBMX, TRMT112, ZCCHC4, CPSF5, CPSF6, CBLL1, SETD2, SRSF3, SRSF10, XRN1, NXF1, PRRC2A, ELAVL1, EIF3A, and EIF3H), and 3 erasers (FTO, ALKBH3, and ALKBH5). For this process, the criteria of |Pearson R| > 0.4 and *p* < 0.001 were used to evaluate the correlation between m6A regulator genes and lncRNAs using Pearson’s correlation analyses.

### Weighted gene co-expression networks analysis

We first extracted a total of 1725 m6A-related lncRNAs for WGCNA by using a “WGCNA” package (Langfelder and Horvath, 2008). When the optimal soft threshold was graphically determined, we then converted the adjacency matrix into the topological overlap matrix (TOM). The modeEigenges function in the “WGCNA” package was used to calculate the dissimilarity of the module eigengenes (MEs). We further estimated the association between MEs and clinical traits by |Pearson R| > 0.2 and *p* < 0.05. Finally, we identified the modules that were significantly associated with the clinical traits for further analyses.

### Consensus clustering based on m6A-related prognostic lncRNAs

According to the expression of 28 m6A-related prognostic lncRNAs (univariate Cox regression analysis, *p* < 0.01), We followed the methods of Wei Song et al. (Song et al., 2021). to clarify the biological characteristics using the R package “ConsensusClusterPlus.” In different clustering subtypes, the principal component analysis (PCA), t-distributed stochastic neighbor embedding (t-SNE), Kaplan–Meier survival analysis, and TME scores were performed.

### Risk model construction and validation of m6A-related prognostic lncRNAs

We screened the prognosis of m6A-related lncRNAs by univariate Cox regression analysis (*p* < 0.01), and then analyzed LASSO Cox regression by using the R package “glmnet” (Xu et al., 2020). The risk score was calculated according to the following: Risk score = ∑Expi * βi (Expi, each lncRNA expression; βi, each lncRNA coefficient). Next, subgroups, including the low-risk group and high-risk group, were established based on the training dataset median risk score. Receiver operating characteristic (ROC) analysis was used to assess the accuracy of the lncRNA signatures in the training dataset, testing dataset, and complete dataset by using the R package “timeROC” (Zhang et al., 2021a; Zeng et al., 2021). Effective dimensionality reduction, model recognition, and grouping visualization of high-dimensional data for the risk model were performed by principal component analysis (PCA) and t-distributed stochastic neighbor embedding (t-SNE).

### Tumor microenvironment cells infiltration based on gene set enrichment analysis and functional enrichment analysis

To explore the potential KEGG pathway related to the m6A-related prognostic lncRNAs, the GSEA method was performed to screen the significant pathways between different clustering subtypes (*p* < 0.05). The infiltration of 22 human immune cell subsets were calculated by the CIBERSORT algorithm for each patients and infiltrative fractions of 22 immune cell types in different clustering subtypes were visualized by using the R package “vioplot” (Li et al., 2021a). TME scores of CRC patients were calculated by using the ESTIMATE algorithm. We also used boxplots to examine the differential expression levels of immune checkpoints between different clustering subtypes. Further, according to the median risk score, we performed the Gene Ontology (GO) and Kyoto Encyclopedia of Genes and Genomes (KEGG) analysis to identify functional comments by applying the R package “clusterProfiler” (*p* < 0.05) in high- and low-risk group (Zhao et al., 2020; Gong et al., 2021). ssGSEA was performed using the R package “GSVA” (Zhang et al., 2021a; Zeng et al., 2021), and immune cell infiltration scores were calculated to evaluate the activity of immune-related pathways.

### Construction of a nomogram for prediction

Based on the lncRNA signatures’ risk score and other clinical predictors, the nomogram prediction model was set up by using the R package “rms” for the 1-year, 3-year, and 5-year OS (Iasonos et al., 2008). The calibration and accuracy of the nomogram were verified by the calibration plot (bootstrap methods with 1,000 replicates).

### Mutation, microsatellite instability, cancer stem cell index, and drug susceptibility analysis

To determine the somatic mutations of CRC patients between high- and low-risk groups, the mutation annotation format (MAF) from the TCGA database was followed by the methods of Xi Zhang et al. based on “maftools” R package (Zhang et al., 2021c). We also calculated the tumor mutation burden (TMB) score for each patient with CRC in the two risk groups. Furthermore, we analyzed the relationships between the two risk groups and microsatellite instability (MSI) and cancer stem cell (CSC) index. To explore differences in the therapeutic effects of chemotherapeutic drugs in patients in the two risk groups, we calculated the semi-inhibitory concentration (IC50) values of chemotherapeutic drugs commonly used to treat CRC followed by the methods of Min Zhou et al. based on the “pRRophetic” package (Zhou et al., 2021).

### Identification and validation of immune genes and ceRNA networks shared by lncRNA signatures

The pearson’s correlation analysis was performed to screen common immune genes shared by 8 lncRNA signatures. The STRING website (https://string-db.org/) was used to predict interactions between functional proteins. The miRcode (http://www.mirco de. org/) database was utilized to predict lncRNA targets interacting with miRNAs. TargetScan (http://www.targe tscan. org/), miRDB (http://www.mirdb.org/miRDB/), and miRTarBase (http://mirtarbase.mbc.nctu.edu.tw) were used to predict the relationship between miRNAs and target mRNAs. The Cytoscape plug-in cytoHubba was performed to identify the hub immune genes. The generated ceRNA networks were visualized by Cytoscape software (version 3.8.2, https://www.cytoscape.org/). The Kaplan-Meier survival analysis was used to compare the survival difference between high- and low-expression of immune genes. To characterize immune genes expression distribution and the heterogeneity in TME of CRC, we search for single-cell sequencing data of CRC from Tumor Immune Single-cell Hub (TISCH) (Sun et al., 2021). The CRC_GSE146771_10X dataset was selected for further analysis.

### Cell culture, real-time PCR and oligonucleotide transfection

The normal intestinal epithelial FHC cells was purchased from ATCC and grown in RPMI-1640 (Gibco, United States). The colon cancer cell lines HCT116 and HT29 were obtained from ATCC and grown in McCoy’s 5A (Gibco, United States). All cells culture medium was supplemented with 10% FBS (Gibco) and 1% penicillin and streptomycin (Beyotime, China) and cultured at 37°C in a humidified 5% CO2 atmosphere. Total RNA was extracted from normal and tumor cell lines by Trizol reagent (Invitrogen, Carlsbad, CA, United States). The total RNA was performed to synthesize complementary DNA (cDNA) by using PrimeScript RT reagent Kit (Takara, Japan). The cDNAs were subjected to SYBR Green assays (Takara) based RT-qPCR on a CFX-96 instrument (Bio-Rad Laboratories). The primers used in real-time PCR assays were listed in Supplementary Table S1. SiRNA was obtained from Genepharma (Shanghai, China) (Supplementary Table S1). Oligonucleotide transfection was performed by using Lipofectamine 3000 (Invitrogen, United States), while nonspecific mRNAs were used as negative controls. The Ct values obtained from different samples were compared using the 2^−ΔΔCT^ method. GAPDH served as internal reference genes and all plasmids were constructed by Shanghai Obio Techonology Company, Shanghai, China.

### Western blot analysis

Total cellular protein was extracted and separated by 10% SDS-PAGE then transferred to polyvinylidene fluoride (PVDF) membranes. Membranes were blocked with 5% non-fat dry milk in PBST for 1 h at room temperature and then incubated with the primary antibodies overnight at 4°C, followed by incubation with secondary antibodies for 2 h at room temperature. Specific protein bands were visualized using the enhanced chemiluminescent (ECL) assay kit (Thermo Scientific, PA, United States). The following primary antibodies were used for western blotting, E-cadherin (3195), N-cadherin (13116), Vimentin (5741), β-catenin (8457). All antibodies were purchased from Cell Signaling.

### Drug susceptibility analysis, cell proliferation assay and drug cytotoxicity assay

Spearman’s correlation analysis was performed to calculate the correlation between key lncRNA signatures expression and chemotherapeutics IC50 values (statistical significance was set at *p* < 0.05). Cell Counting Kit-8 (CCK8; Yeasen, China) was performed to assay the percentage of viable cells based on different treatment conditions. Firstly, cells were seeded in 96-well plates with 100 ul culture medium. Secondly, the 10 ul of CCK-8 solution was added to each well at specific time points and incubated at 37°C for 4 h. The reaction product was measured according to the manufacturer’s protocol.

### Statistical analysis

All statistical analyses were performed using R software (v4.1.0), GraphPad Prism (version 9.0), and SPSS software (23.0). To compare the gene expression levels, we applied the single-factor analysis of variance, while the categorical variables were determined using the Pearson chi‐square test. Kaplan–Meier curve analyses were used to identify the survival differences between different groups. The independent prognostic value of the risk model was assessed by using the univariate and multivariate Cox regression models. The Mann–Whitney test and an independent *t*-test were used to compare the differences between different groups. *p* < 0.05 was considered statistically significant for all analysis results.

## Results

### Identification of m6A-related lncRNAs in colorectal cancer patients

**FIGURE 1:**
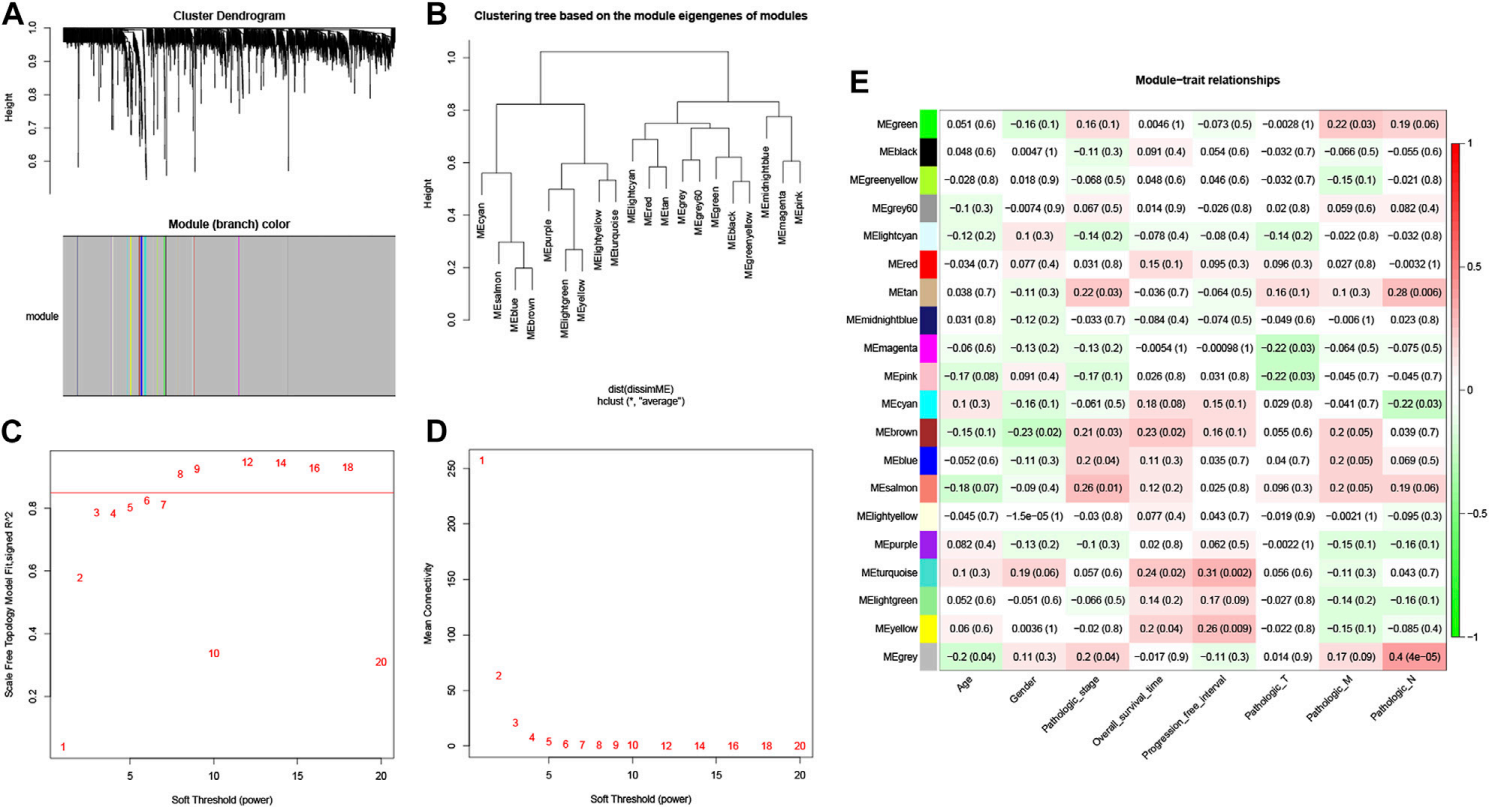
Weighted co-expression network analysis. **(A)** Hierarchical clustering dendrograms of identified co-expressed m6A-related lncRNAs in modules of CRC. **(B)** cluster analysis of samples dendrogram. Analysis of the scale-free fit index **(C)** and the mean connectivity **(D)** for various soft-thresholding powers. **(E)** Heatmaps of the module-trait associations were evaluated by the correlation and *p* value between module eigengenes and sample traits.

To identify potential m6A-related lncRNAs, we evaluated the relationship between 39 m6A methylation regulators and all lncRNAs *via* combined Pearson correlation analysis and Weighted gene co-expression network analysis (WGCNA). We first identified 13,185 interactions and 1,725 m6A-related lncRNAs with │Pearson R│> 0.4 and a *p* < 0.001. As is known, WGCNA is a network analysis method used to identify co-expression modules based on the correlation coefficient. In addition, it is applied to multi-sample data, with >15 samples (Langfelder and Horvath, 2008) usually required. To make the results better and more reliable, the number of samples usually required is > 20 (24). Therefore, we randomly selected 100 samples to construct the gene co-expression network. We then calculated the 1,725 m6A-related lncRNAs associated with clinical traits, including age, gender, the pathologic stage, overall survival (OS) time, progression-free interval (PFI), and TMN stages. In our study, the parameters were established by setting the soft threshold power to 6 (scalefree *R* ^2^ = 0.8) and the height to 0.89, and 20 modules were identified (Figures 1A–D). The correlation between module eigengene (ME) values and clinical traits was measured to determine the association between the modules and clinical characteristics. The results in Figure 1E show that a total of 11 modules were closely correlated with clinical characteristics (│Pearson R│> 0.2 and a *p* < 0.05). For example, the green, tan, magenta, pink, cyan, brown, blue, salmon, and grey modules were significantly associated with tumor progression. The brown, turquoise, and yellow modules were significantly associated with prognosis. The brown and grey modules were significantly associated with age and gender. These results indicate that each module might represent the specific clinical characteristics and potential biological significance of CRC patients. Thus, these 11 modules with a total of 1,707 m6A-related lncRNAs were selected to further explore the correlation between the progression and prognosis of CRC patients.

### Consensus clustering analysis of m6A-related prognostic lncRNAs in colorectal cancer patients

**FIGURE 2:**
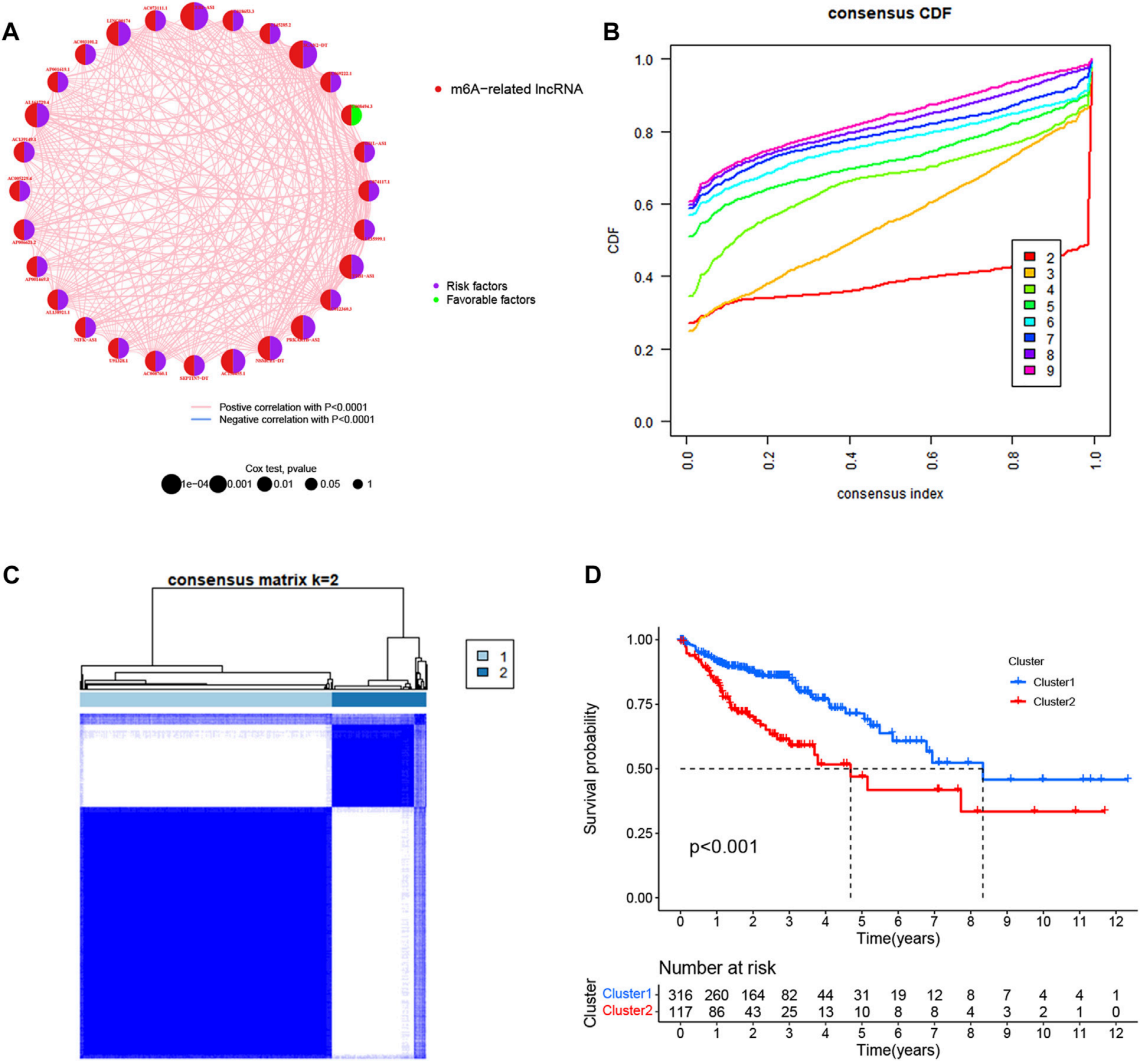
Identification of m6A-related prognostic lncRNAs. **(A)** Univariate cox regression analysis of OS for 28 m6A-related prognostic lncRNAs in TCGA cohorts. **(B)** Consensus clustering cumulative distribution function (CDF) for *k* = 2 to 9. **(C)** Consensus clustering matrix for *k* = 2. **(D)** Kaplan–Meier OS curves for the two clusters.

**FIGURE 3:**
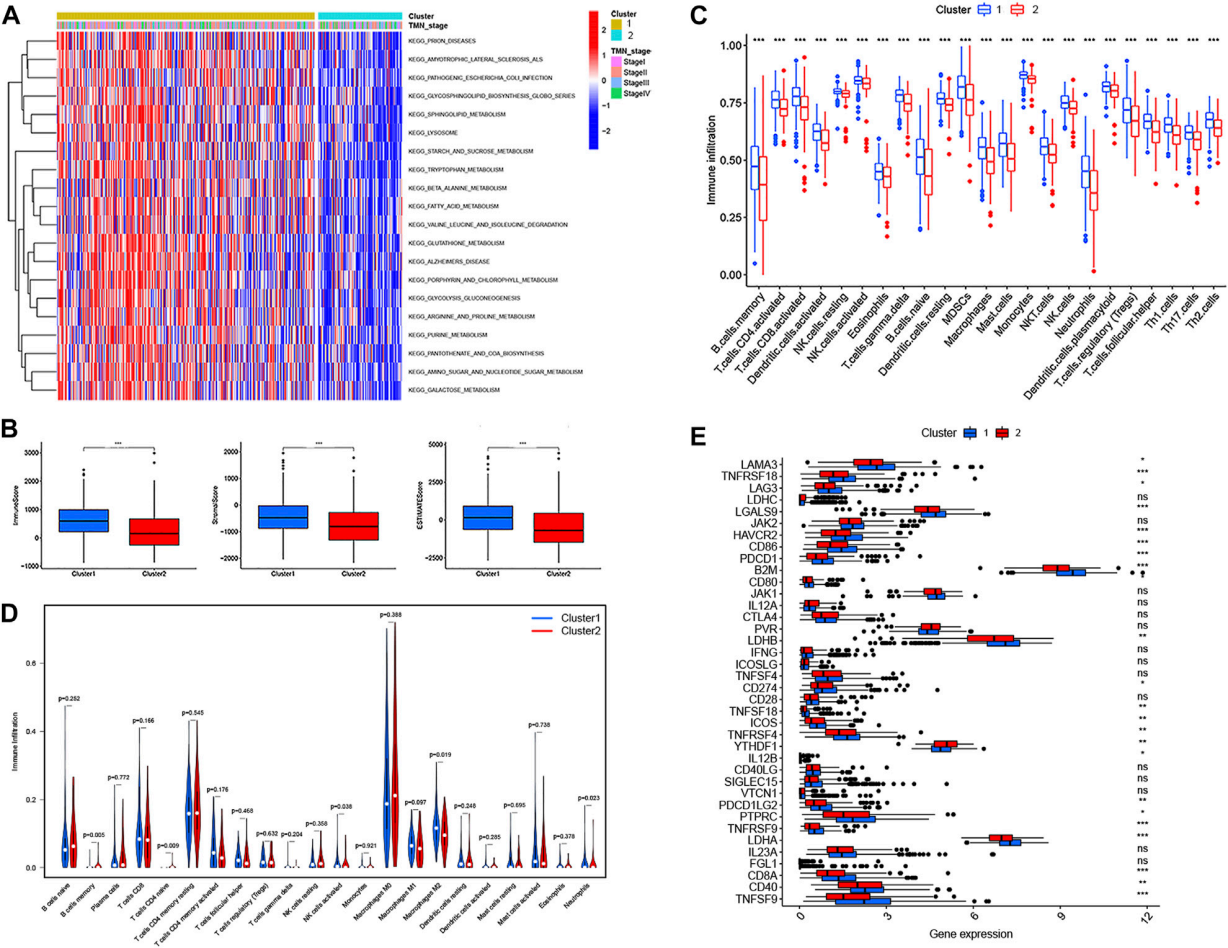
TME infiltration and immune-oncology targets in cluster 1/2 subtypes constructed by 28 m6A-related prognostic lncRNAs. **(A)** Biological processes analyzed by GSVA which showed the active biological pathways in distinct subtypes. **(B)** The variance analysis of ImmuneScore, StromalScore, and ESTIMATEScore in cluster 1/2 subtypes. **(C)** The abundance of 22 TME infiltrating cells between the two CRC subtypes by ssGSEAScore. **(D)** The differential infiltration levels of 22 kinds of immune cells in cluster 1/2 subtypes based on the CIBERSORT platform. **(E)** Expression of immune checkpoints in cluster 1/2 subtypes. *p < 0.05; **p < 0.01; ***p < 0.001.

We screened the prognostic lncRNAs derived from the 1,707 m6A-related lncRNAs using univariate Cox regression analysis (*p* < 0.01). A total of 28 m6A-related prognostic lncRNAs in the TCGA dataset were significantly correlated with overall survival. Among them, 27 m6A-related prognostic lncRNAs (AC074117.1, ASH1L−AS1, AC069222.1, ZKSCAN2−DT, AC018653.3, ZEB1−AS1, AC073111.1, LINC00174, AC003101.2, AP001619.1, AL161729.4, AC139149.1, AC005229.4, AP006621.2, AP001469.3, AL138921.1, AC008760.1, AC156455.1, NSMCE1−DT, AC012360.3, AL135999.1, AC145285.2, NIFK−AS1, U91328.1, SEPTIN7−DT, PRKAR1B−AS2, ATP2B1−AS1) were risk factors with a hazard ratio >1, while 1 m6A-related prognostic lncRNA (AC008494.3) was a protective factor with a hazard ratio <1 (Figure 2A). To further clarify the correlation between these 28 m6A-related lncRNAs’ expression character and OS in patients, we performed consensus clustering analysis to identify CRC prognosis subtypes based on these lncRNAs. Results showed that the patients were separated into two different subtypes according to the optimal clustering stability value (*k* = 2), with 316 cases included in cluster 1 and 117 cases included in cluster 2 (Figures 2B,C). The overall survival of cluster 2 for CRC patients was worse than that of cluster 1 (*p* < 0.001) (Figure 2D). GSVA enrichment analysis was performed to identify the differences in biological behavior between these two subtypes. As shown in Figure 3A, cluster 1 was significantly enriched in intestinal pathogen infection, sphingolipid metabolism, the functions of lysosome, tryptophan and fatty acid metabolism, amino sugar and nucleotide sugar metabolism, glutathione and galactose metabolism. Further, Gene Set Enrichment Analysis (GSEA) was used to elucidate the potential regulatory mechanisms leading to the differences between the two clusters of CRC patients. As shown in Supplementary Figures S2A,S2B, we discovered that patients in cluster 1 were mainly enriched with citrate cycle TCA, oxidative phosphorylation, the functions of proteasomes and peroxisomes, amino sugar and nucleotide sugar metabolism, and adhesion junctions, the Wnt signaling pathway, dorso-ventral axis formation, taste transduction, and basal cell carcinoma were involved in cluster 2.

### Two m6A-related prognostic lncRNA subtypes associated with tumor microenvironment infiltration, immune-oncology targets, and clinical characteristics

**FIGURE 4:**
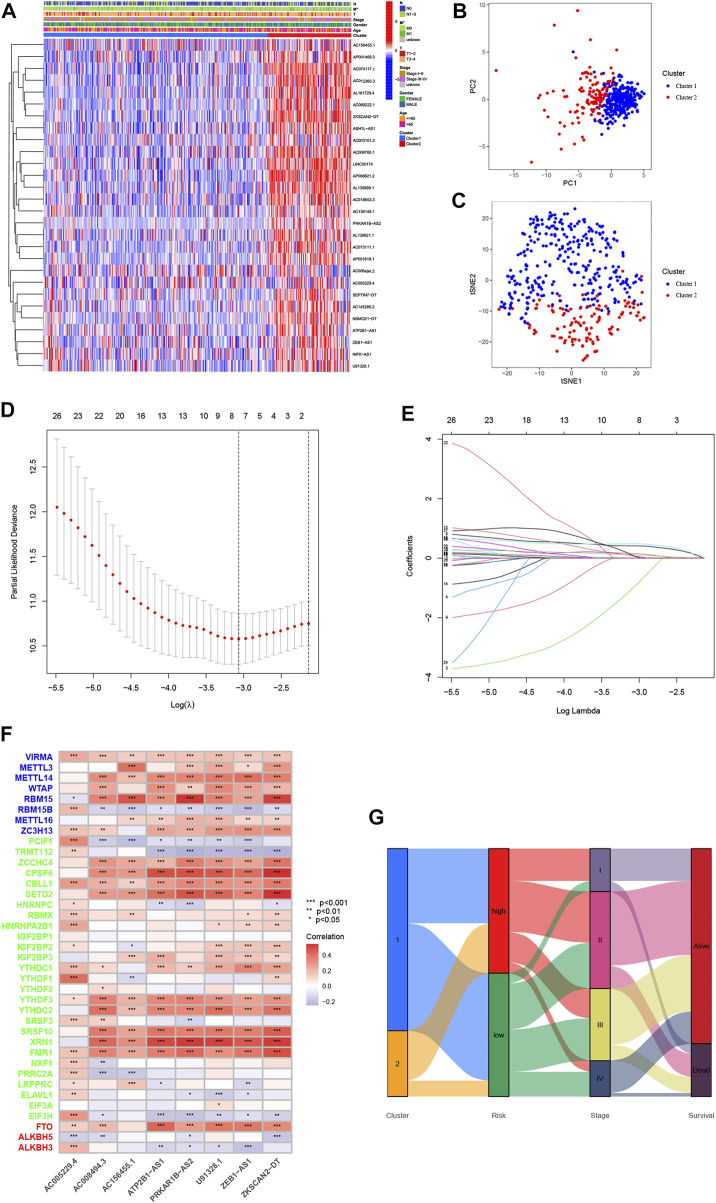
Characterization of cluster 1/2 subtypes and construction of m6A-related lncRNA signatures based on 28 m6A-related lncRNAs. **(A)** Heatmap of the associations between the cluster 1/2 subtypes and clinical characteristics. **(B,C)** The cluster 1/2 subtypes based on 28 m6A-related prognostic lncRNAs were performed to Principal Component Analysis **(B)** and t-Distributed Stochastic Neighbor Embedding **(C)** analysis. **(D)** Plots of the minimum 10-fold cross-validation to select the tuning parameter in the LASSO regression. **(E)** The LASSO coefficient of 28 m6A-related prognostic lncRNAs. **(F)** Heatmap for the correlations between all m6 A genes and the 8 prognostic m6A-related lncRNAs. **(G)** Alluvial diagram of subtype distributions in groups with different m6A-related lncRNA signatures and survival outcomes. *p < 0.05; **p < 0.01; ***p < 0.001.

To further investigate the correlation with TME infiltration, we then analyzed the differences in terms of ImmuneScore, StromalScore, and EstimateScore between cluster 1 and cluster 2. Our results revealed that cluster 2 had a lower ImmuneScore and StromalScore than cluster 1, which might indicate that cluster 2 had a higher tumor purity (Figure 3B). According to the CIBERSORT algorithm, we observed that the score of 22 TME infiltrating cells were all significantly poorly activated in cluster 2 by ssGSEAScore (Figure 3C). In addition, we found that the abundance of 22 TME infiltrating cells were significantly differentially expressed in two subtypes. For example, innate immune cell infiltration was abundant in cluster 1, including the presence of M2 macrophages, NK cells, and neutrophils cells, while B memory cells and CD4^+^ T naive cells were enriched in cluster 2 (*p* < 0.05) (Figure 3D). Therefore, this was consistent with the fact that the ImmuneScore of cluster 1 was higher than that of cluster 2. Furthermore, we investigated the associations between immune checkpoints and the two subtypes. Figure 3E shows that 38 immune checkpoints were differentially expressed between cluster 1 and cluster 2, including PD-1, PD-L1, PD-L2, and CTLA-4. Finally, the expression profile of 28 m6A-related prognostic lncRNAs and their association with clinical characteristics, including age, gender, the pathologic stage, and TMN stages, was presented in a heatmap. We found that the M classification of TMN stages was significantly different between the two subtypes (*p* < 0.05) (Figure 4A). In addition, we also demonstrated that the two different subtypes could be well distinguished by principal component analysis (PCA) and t-distributed stochastic neighbor embedding (t-SNE) analysis (Figures 4B,C). Taken together, our results indicated that the 28 m6A-related prognostic lncRNAs were involved in shaping the TME, and represented different prognostic characteristics in CRC patients.

### LncRNA signature construction and validation of a risk model in colorectal cancer patients

**FIGURE 5:**
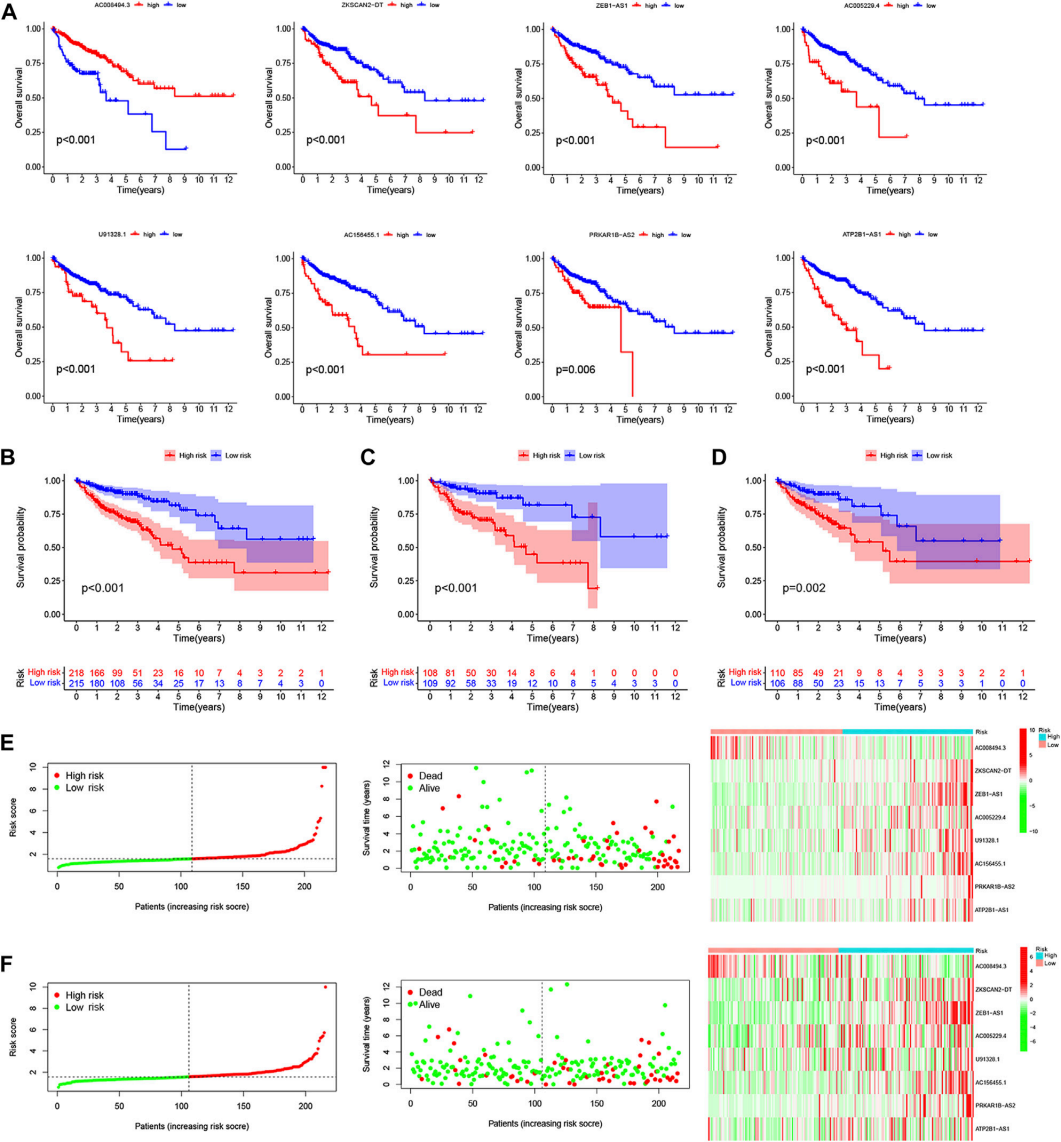
Construction and validation of a risk model based on the 8 m6A-related lncRNA signatures. The OS curve of 8 m6A-related lncRNA signatures for the low- and high-expression groups with the cut-off value 0.599 from TCGA cohorts **(A)**. Kaplan-Meier survival curves of the OS for patients in the complete dataset **(B)**, training dataset **(C)**, and testing dataset **(D)** between the high- and low-risk group. The distribution of the risk score, survival time and survival status, and the heatmap of the 8 m6A-related lncRNA signatures in the training dataset **(E)**, testing dataset **(F)** between the high- and low-risk group.

**FIGURE 6:**
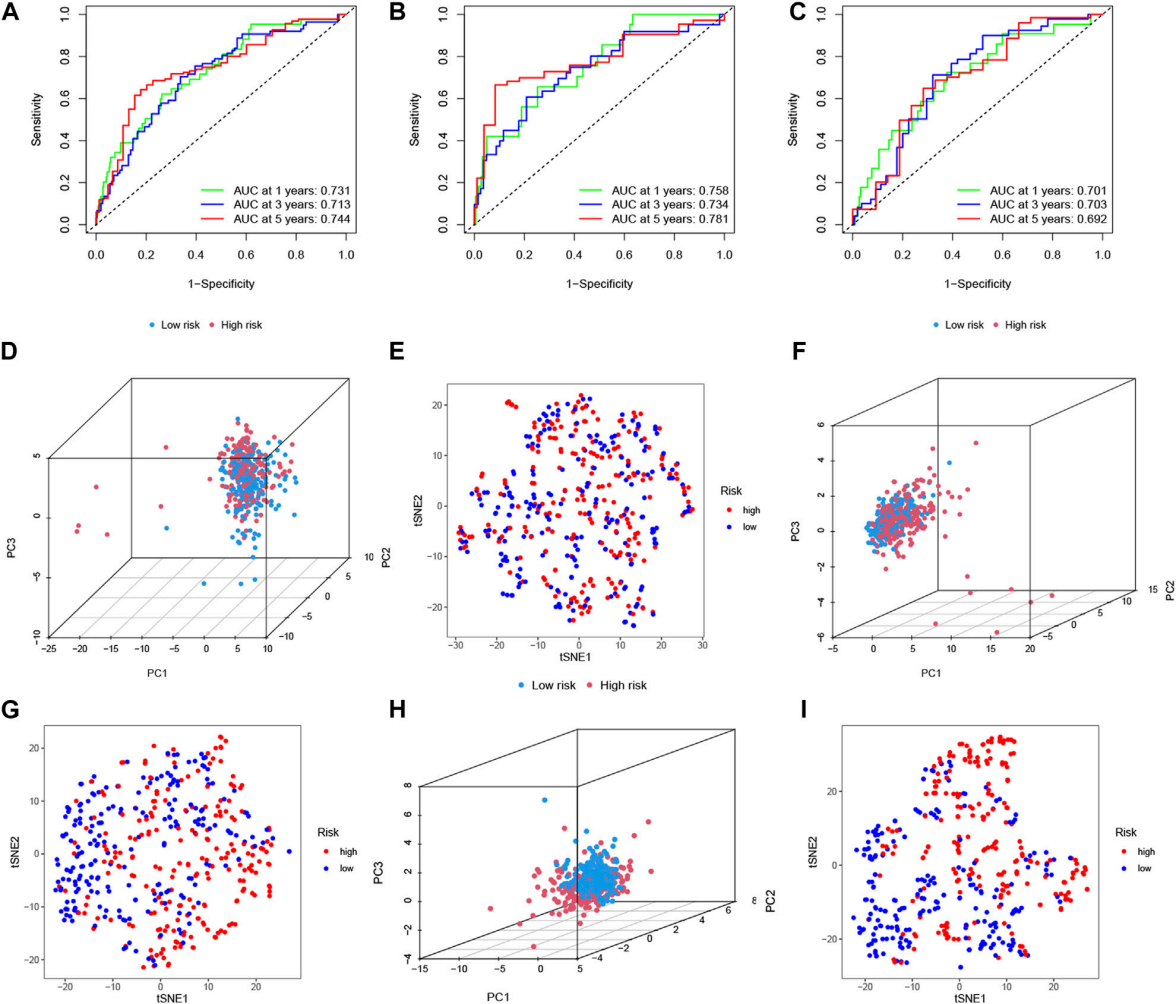
ROC, PCA, and t-SNE analysis between the high- and low-risk group. The 1-, 3-, and 5-years ROC curves of 8 m6A-related lncRNA signatures for the OS prediction in the complete dataset **(A)**, training **(B)**, and testing **(C)**. Principal component analysis (PCA) between the high- and low-risk group based on **(D)** 39 m6A methylation regulators, **(F)** 28 m6A-related prognostic lncRNAs, and **(H)** 8 m6A-related lncRNA signatures. t-Distributed Stochastic Neighbor Embedding (t-SNE) analysis between the high- and low-risk group based on **(E)** 39 m6A methylation regulators, **(G)** 28 m6A-related prognostic lncRNAs, and **(I)** 8 m6A-related lncRNA signatures.

To clarify the biological function of m6A-related lncRNAs, we further explored the prognostic value of CRC patients by merging the expression and clinical data. A total of 433 patient samples were divided into the training dataset (*N* = 217) and the test dataset (N = 216). We then performed a LASSO penalized Cox regression analysis to construct an 8 m6A-associated lncRNA signatures model using the training dataset (Figures 4D,E). Therefore, the risk score for each patient in the training dataset, testing dataset, and complete dataset was calculated based on the risk formula: risk score = AC008494.3 * (−0.964259730717564) + ZKSCAN2-DT * 0.010056256050767 + ZEB1-AS1 * 0.413400864759708 + AC005229.4 * 0.154898466270611 + U91328.1 * 0.077293286865632 + AC156455.1 * 0.112691335331562 + PRKAR1B-AS2 * 0.485291921513758 + ATP2B1-AS1 * 0.0706027766158002. The correlation between m6A genes and 8 m6A-related lncRNA signatures was shown in Figure 4F. An alluvial diagram was used to visualize the distribution of patients in the two subtypes and two risk score groups (Figure 4G). Moreover, survival curves showed that all the 8 m6A-related lncRNA signatures were survival-associated with OS (Figure 5A). Further, the three datasets were categorized into low- and high-risk groups based on the training dataset median value of the prognostic risk grade. The results of Kaplan–Meier (KM) curve analysis showed that the low-risk group had a better prognosis than the high-risk group in the complete, training and testing datasets (*p* < 0.05) (Figures 5B–D). The correlation between the 8 lncRNA signatures and the risk score can be observed in the heatmap, and the distribution of the risk score and survival time of CRC patients is displayed in the training and testing datasets (Figures 5E,F). These results shown that the expression levels of ZKSCAN2−DT (HR: 1.421, 95%CI: 1.218–1.657), ZEB1−AS1 (HR: 2.125, 95%CI: 1.495–3.021), AC005229.4 (HR: 1.481, 95%CI: 1.101–1.992), U91328.1 (HR: 2.30, 95%CI: 1.339–3.951), AC156455.1 (HR: 1.178, 95%CI: 1.074–1.293), PRKAR1B−AS2 (HR: 2.092, 95%CI: 1.424–3.073), and ATP2B1−AS1 (HR: 7.787, 95%CI: 2.55–23.782) were higher, while the expression levels of AC008494.3 (HR: 0.129, 95%CI: 0.027–0.605) was lower in the high-risk group than in the low-risk group. In addition, the area under the curve (AUC) values for 5-year OS were 0.74, 0.78, and 0.69 in the complete, training and testing datasets, respectively (Figures 6A–C). Interestingly, compared with others’ risk models (0.62 and 0.67) (Zhang et al., 2021a; Zeng et al., 2021), our testing datasets’ AUC values (5-year) for the risk signature are the highest, and the AUC of the risk score was also higher than the AUCs of other clinicopathological characteristics (Supplementary Figure S3A), indicating that our signature is a reliable prognostic model. To further predict the ability of the prognostic model, the disease-free interval (DFI), progression-free interval (PFI), and disease-specific survival (DSS) were investigated to distinguish high- and low-risk in CRC patients. As predicted, the DFI, PFI, and DSS differed between the low- and high-risk group, implying that the lncRNA signatures had good accuracy in the prognostic prediction of CRC (*p* < 0.05) (Supplementary Figures S3B–S3D). Subsequently, the PCA and t-SNE were performed to test the difference between the low- and high-risk groups based on the 39 m6A methylation regulators, 28 m6A-related lncRNAs, and 8 lncRNA signatures. Notably, our results suggested that the low- and high-risk group were better distinguished by the 8 lncRNA signatures than the 39 m6A genes as well as the 28 m6A-related lncRNAs (Figures 6D–I).

### Stratification analysis and independent prognostic value of the lncRNA signatures

**FIGURE 7:**
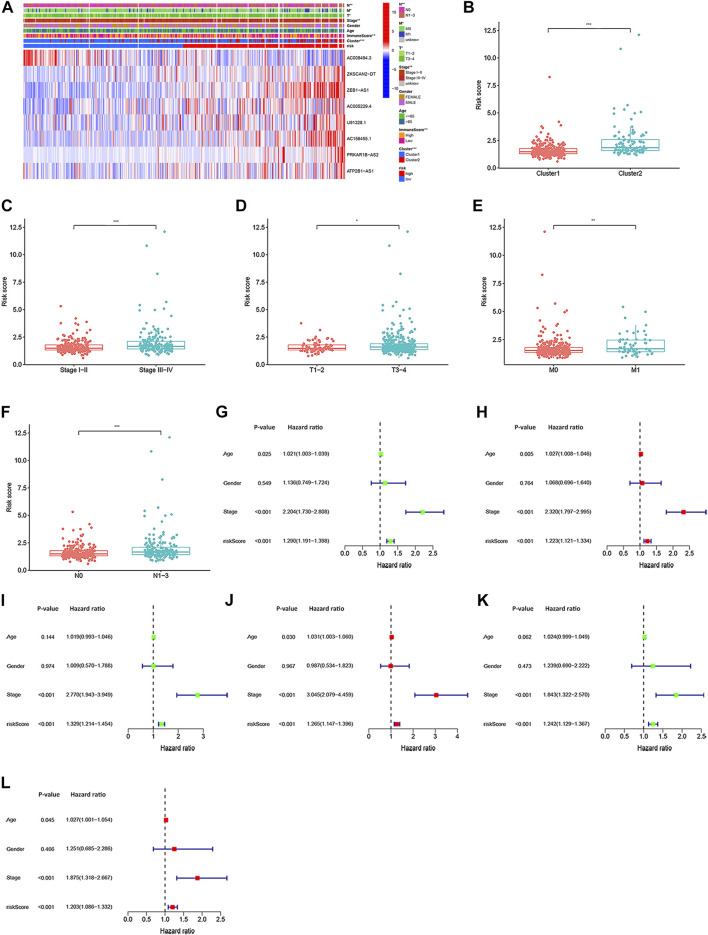
The association of risk score with clinical characteristics, and univariate and multivariate Cox regression analyses for the risk score based on the 8 m6A-related lncRNA signatures. **(A)** Heatmap of the correlation of 8 m6A-related lncRNA signatures with cluster 1/2 subtypes, immuneScore, and clinical characteristics. Different levels of risk score associated with pathologic-stage **(B)**, TMN staging **(C–E)**, and clusters **(F)**. Univariate Cox regression analysis and multivariate Cox regression analyses for the complete dataset **(G,H)**, respectively), training dataset **[(I,J)**, respectively] and testing dataset **[(K,L)**, respectively]. *p < 0.05; **p < 0.01; ***p < 0.001.

The heatmap was performed to show the expression levels of lncRNA signatures in the high- and low-risk group (Figure 7A). Moreover, the heatmap also demonstrated the significant differences in terms of the pathologic stage (*p* < 0.01), two subtypes (*p* < 0.001), TMN stages (*p* < 0.05) and immuneScore (*p* < 0.01) between the high- and low-risk group. Specifically, we found that the risk score increased significantly from Stage I-II to Stage III-IV. The TMN stages also presented a significant divergence from the T1-2, M0, N0 classification to T3-4, M1, N1-3 classification. Moreover, CRC patients in cluster 2 were associated with a higher risk score than that of patients in cluster 1 (Figures 7B–F). After investigating the association between the lncRNA signatures and clinical factors in CRC patients, as shown in Supplementary Figures S4A–S4F we found that CRC patients in the high-risk group tended to have a lower overall survival rate than the low-risk group in our different stratifications, including age, gender, pathologic stage, and TMN stages. These results suggest that m6A-related lncRNA signatures can predict the prognosis of CRC regardless of clinical factors. Furthermore, univariate and multivariate Cox regression analyses were used to investigate whether the lncRNA signatures could be used as independent prognostic factors in the training, testing, and complete datasets. Through analyses, we found that the lncRNA signatures could act as independent predictors for the prognosis of CRC (Figures 7G–7L).

### Construction of a nomogram for the individualized prediction model

**FIGURE 8:**
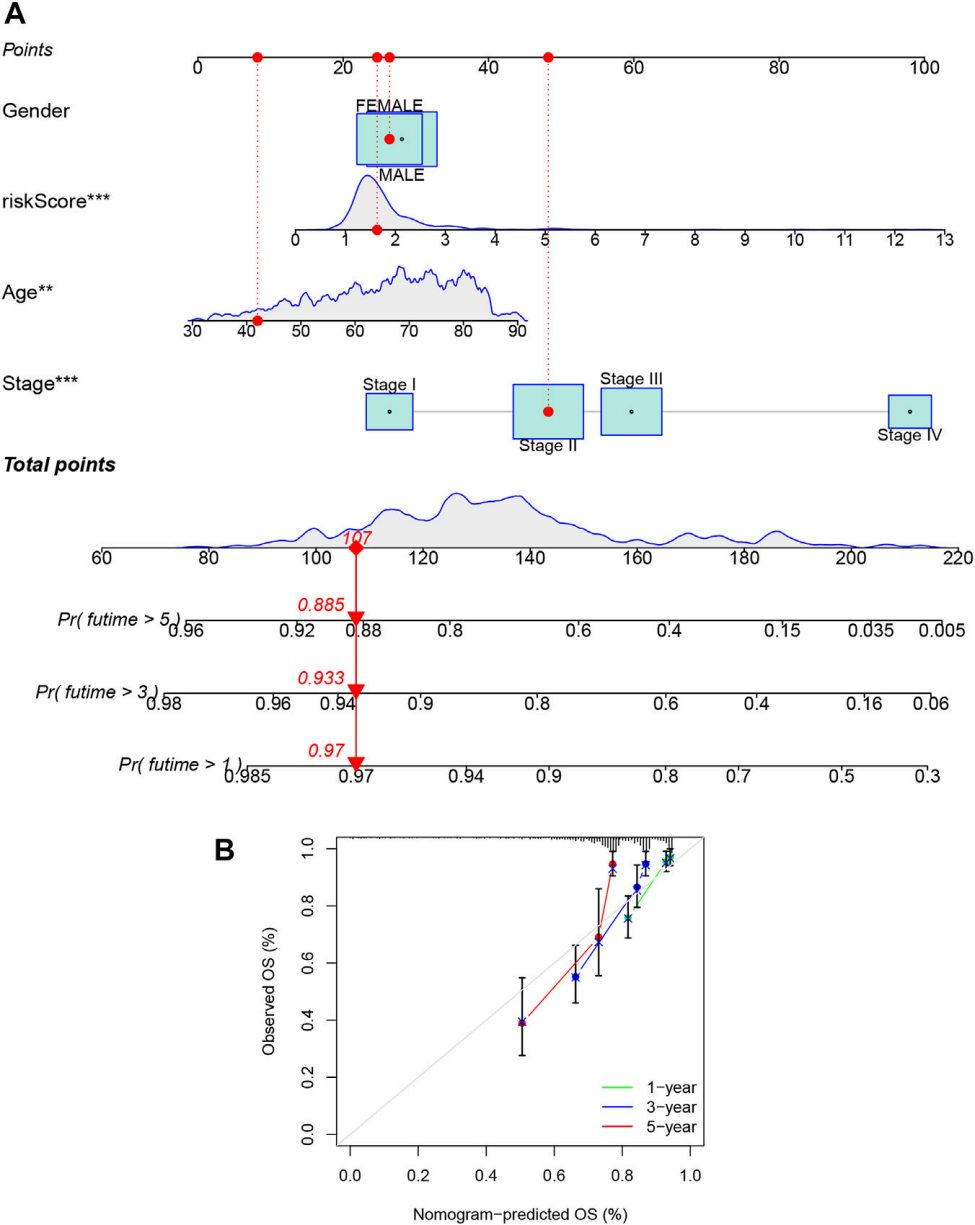
Construction and evaluation of a predictive nomogram. **(A)** The nomogram predicts the probability of overall survival at the 1-, 3-, and 5-year. **(B)** The calibration plot of the nomogram predicts the probability of the 1-, 3-, and 5-year overall survival. *p < 0.05; **p < 0.01; ***p < 0.001.

Given the importance of the lncRNA signatures in predicting the prognosis of CRC patients, we further attempted to construct a nomogram based on the multivariate cox regression for predicting the OS at 1-, 3-, and 5- years. As shown in Figure 8A, the predominant predictive ability of the risk score in the nomogram was exhibited compared with the clinical characteristics, including age, gender, and stage. Moreover, the calibration plots indicated that the 1-, 3-, and 5- year OS rates could be predicted relatively well in the complete dataset (Figure 8B).

### Relationship of lncRNA signatures with tumor mutation burden, microsatellite instability and cancer stem cell index

**FIGURE 9:**
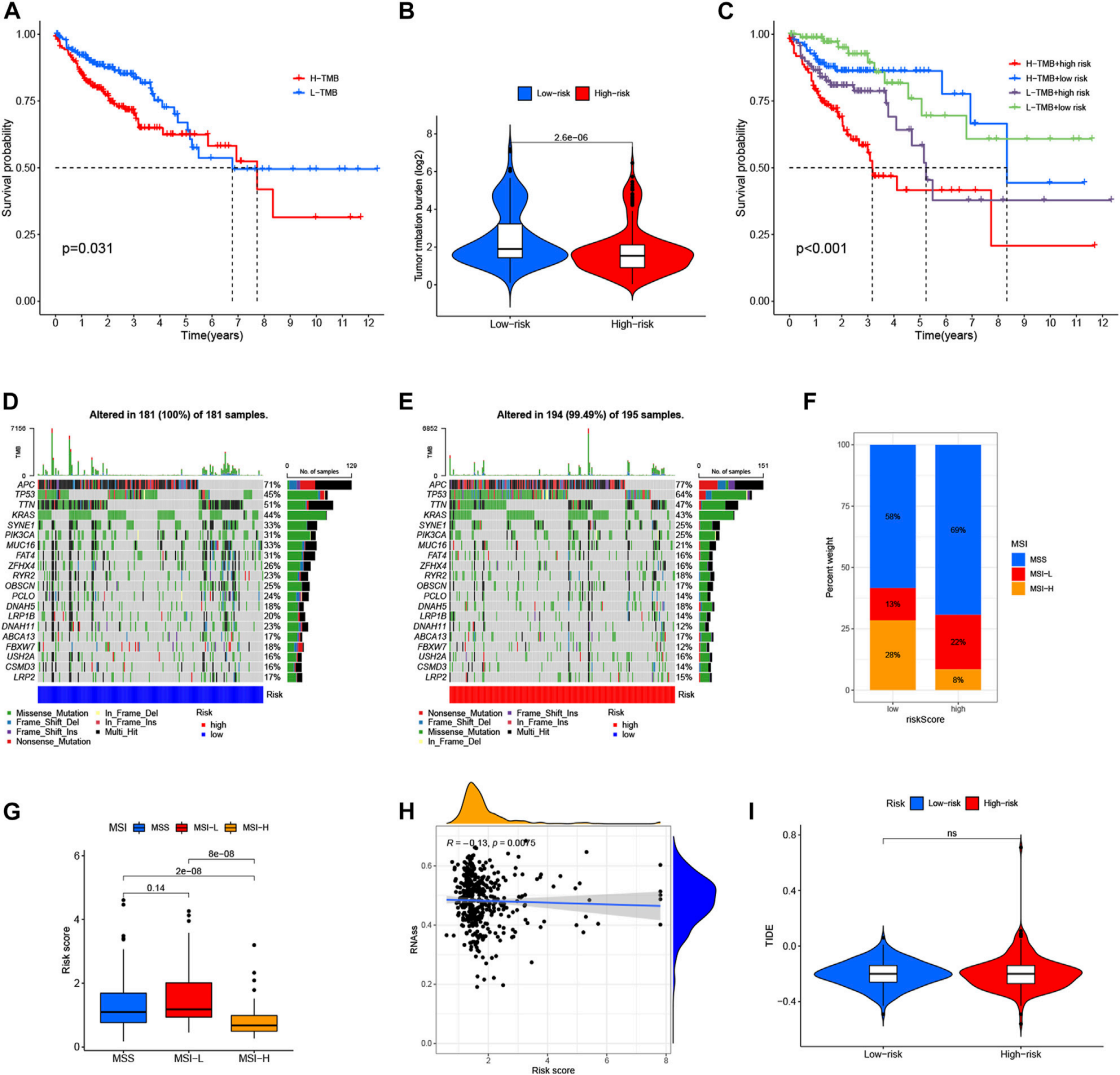
Comprehensive analysis of the m6A-related lncRNA signatures in CRC. **(A)** Kaplan–Meier curves for the high- and low-TMB with the cut-off value 2.711 of 433 colon cancer patients in TCGA cohorts. **(B)** TMB difference in the high- and low-risk groups. **(C)** Kaplan–Meier curves for CRC patients in TMB and risk score subgroups. **(D,E)** The waterfall plot of somatic mutation features established with the high- and low-risk groups. **(F,G)** The correlation between two-risk groups and MSI. **(H)** The correlation between two-risk groups and CSC index. **(I)** The relationship between two risk groups and TIDE.

Based on the TCGA-COAD mutation data, we first assessed the potential relationship between the TMB level and OS, and observed that the survival rate of patients with high TMB was lower than that of patients with low TMB (the optimal cut-off = 2.711, *p* = 0.031, HR: 1.598, 95%CI: 1.041–2.453) (Figure 9A). In addition, Figure 9B showed a higher TMB in the low-risk groups than that in high-risk groups, and combination of the lncRNA signatures risk model and TMB (respectively defined as H-TMB + high-risk, H-TMB + low-risk, L-TMB + high-risk, and L-TMB + low-risk) could clearly stratify patients better (the optimal cut-off = 2.711, *p* < 0.001, HR: 2.205, 95%CI: 1.416–3.435) (Figure 9C). Interestingly, patients with L-TMB + high-risk group had worse survival outcomes than patients with H-TMB + low-risk, indicating that the trend of survival advantage in the L-TMB group was reversed by the risk score. Further, the distribution variations of the top ten somatic mutations genes between two risk signature models were analyzed based on the TCGA-COAD cohort (Figures 9D,E). Except for the APC and TP53, other mutated genes were higher mutations frequencies in the low-risk groups compared to the high-risk groups. Moreover, correlation analyses revealed that the high-risk groups were associated with microsatellite stable (MSS) and low microsatellite instability (MSI−L) status, while low-risk groups were correlated with high microsatellite instability (MSI-H) status (Figures 9F,G). In addition, we analyzed the relationships between risk score and cancer stem cell (CSC) in the two risk groups. As shown in Figure 9H, the result demonstrated a negative correlation between risk score and the CSC index values (R = −0.13, *p* < 0.01), implying that the low-risk score had more distinct stem cell properties and a lower degree of cell differentiation.

### lncRNA signatures predict sensitivity of colorectal cancer to immunotherapy and chemotherapy

There is increasing evidence that patients with a high TMB or MSI-H status may benefit from PD-L1 treatment (Snyder et al., 2014; Topalian et al., 2016; Picard et al., 2020). However, we found there was no difference in dysfunction and exclusion score by using the Tumor Immune Dysfunction and Exclusion (TIDE) algorithm between the two risk groups (Figure 9I), which was inconsistent with previous studies, and might be related to the fact that there were no statistical differences in immune checkpoints between high- and low-risk groups in our study (Supplementary Figure S3F). In addition, CRC recurrence is attributed to chemoresistance. Thus, we further performed to analysis the drug-sensitivity (IC50 value) that currently were used for the treatment of CRC (such as shikonin, cisplatin, gemcitabine, paclitaxel, gefitinib, sorafenib, pazopanib and camptothecin) by using the “pRRophetic” package. Interestingly, we found that the patients in the high-risk group had lower IC50 values for shikonin and pazopanib, while IC50 values of cisplatin and sorafenib were significantly lower in the patients with low-risk group (Supplementary Figure S5). Moreover, we found that the IC50 values of most existing drugs, such as bortezomib, cytarabine, and metformin, were significantly lower in low-risk groups than those in high-risk groups, while IC50 values of bleomycin, imatinib, midostaurin, nilotinib, and so on were significantly lower in the patients with high-risk groups. Together, these results showed that ours lncRNA signatures were associated with drug sensitivity, and might provide therapeutic schedules for further analysis.

### The correlation between lncRNA signatures and distinct tumor microenvironment infiltration

**FIGURE 10:**
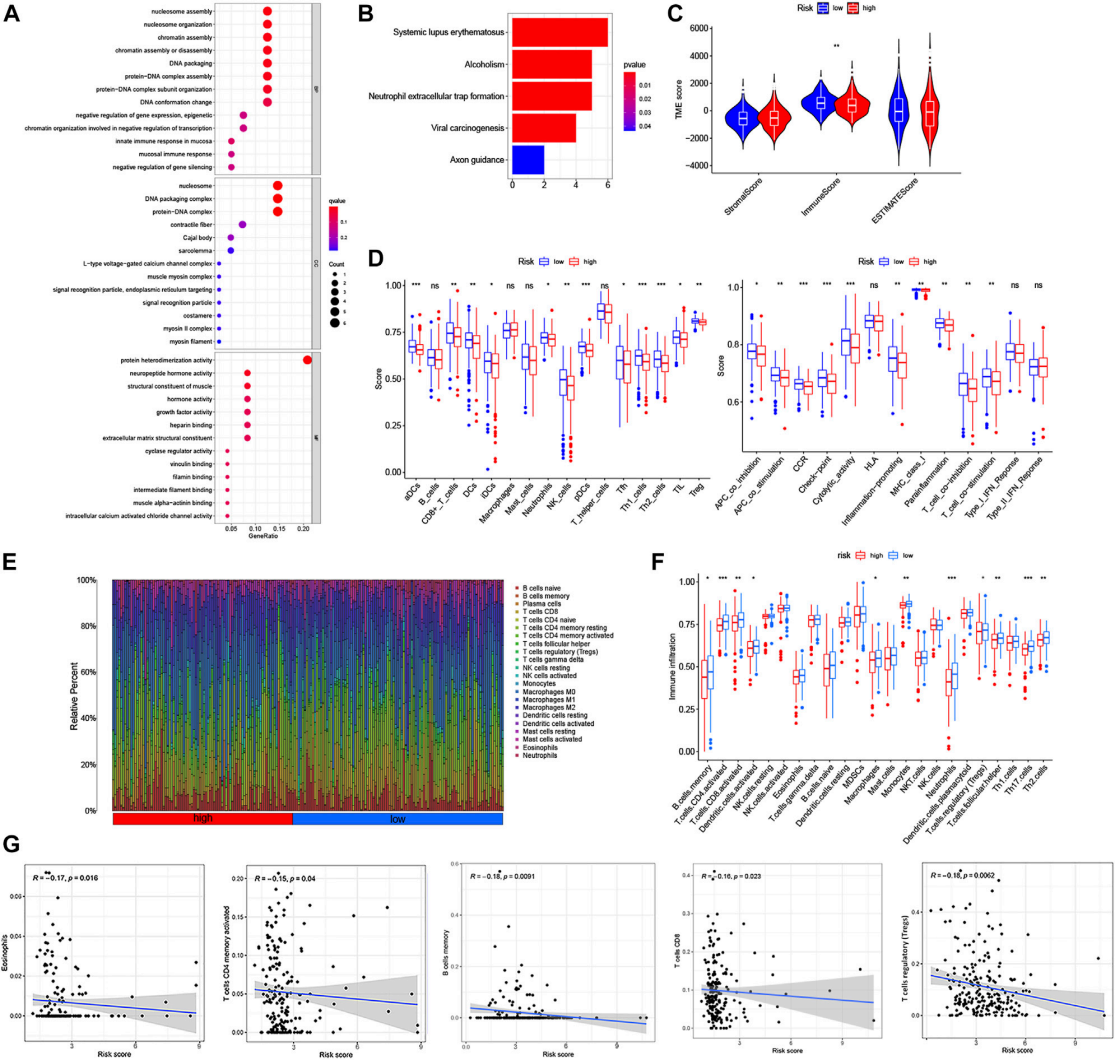
Functional enrichment analysis and comparison of the immune activity between high- and low-risk group based on the 8 m6A-related lncRNA signatures. **(A)** The Bubble graph for gene ontology (GO) analysis. **(B)** The Barplot graph for Kyoto Encylopedia of Genes and Genomes (KEGG) analysis. **(C)** Correlations between the two-risk groups and TME score. **(D)** Comparison of the score of immune cells and immune-related pathways between the high-and low-risk group by ssGSEAScore. **(E)** Barplot showing the proportion of 22 kinds of tumor-infiltrating immune cells in CRC. Column names of plot were sample ID. **(F)** The differential infiltration levels of 22 kinds of immune cells between the two-risk groups based on the CIBERSORT platform. **(G)** The association of risk score with Eosinophil cells, B cells memory, CD8+T cells, CD4 memory-activated T cells and regulatory T (Treg) cells. *p < 0.05; **p < 0.01; ***p < 0.001.

**FIGURE 11:**
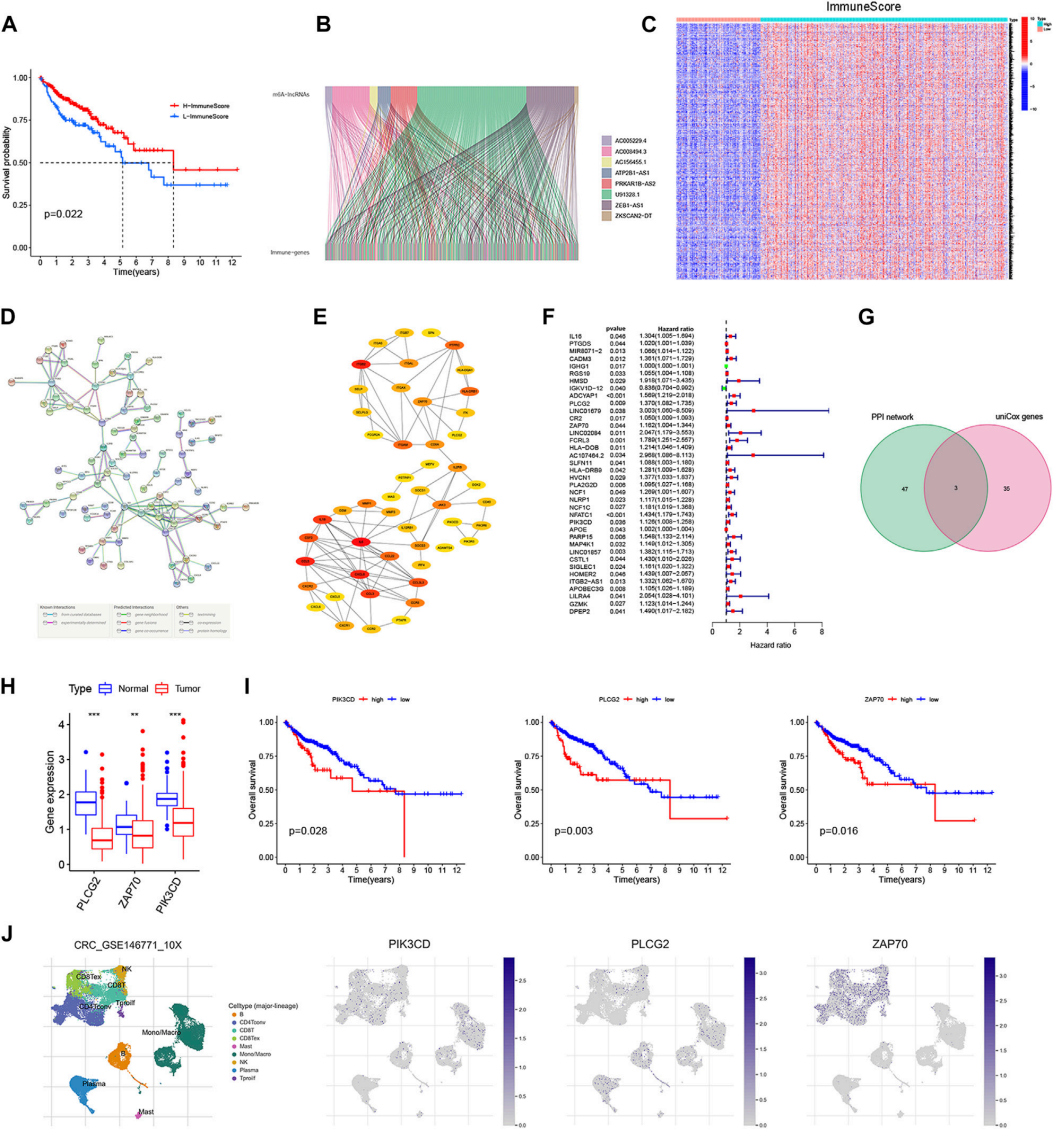
Expression distribution of the 3 immune genes in TME of CRC. **(A)** Kaplan–Meier curves for the high- and low-immunescore with the cut-off value 56.042 of 441 CRC patients. **(B)** Sankey relational diagram for 8 m6A-related lncRNA signatures and DEIGs. **(C)** Heatmap for DEIGs generated by comparison of the high score group vs the low score group in ImmuneScore (Row name of heatmap is the gene name, and column name is the ID of samples which not shown in plot). **(D)** PPI network showing the interactions of DEIGs (the minimum required interaction score was 0.9). **(E)** Fifty hub immune genes in this network with a score of >2. **(F)** Univariate Cox regression analysis with 458 DEIGs, listing the significant factors with *p* < 0.05. **(G)** Venn plot showing the common immune genes shared by leading 50 nodes in PPI and significant factors in univariate Cox. **(H)** Differentiated expression of 3 immune genes in the normal and tumor sample. **(I)** The OS curve of 3 immune genes for the low- and high-expression groups with the cut-off value 2.896. **(J)** The association between the expression of PIK3CD, PLCG2 and ZAP70 and the abundance of tumor-infiltrating immune cells in CRC based on TISCH. **p < 0.01; ***p < 0.001.

To explore the interactions between lncRNA signatures and the TME of CRC, we further employed Gene Ontology (GO) enrichment analysis and Kyoto Encyclopedia of Genes and Genomes (KEGG) pathway analysis to determine differences between the low- and high-risk group using the risk model. The results indicated that the lncRNA signatures were mainly correlated with the immune response, nucleosome assembly, protein heterodimerization activity, systemic lupus erythematosus, and neutrophil extracellular trap formation (Figures 10A,B). Notably, the low-risk groups were closely associated with a high ImmuneScore, whereas the stromal score was no obvious differences in two risk groups (Figure 10C). In addition, we compared the enrichment scores of the immune cells and the immune-related pathways between the low- and high-risk group in CRC patients based on the lncRNA signatures. As shown in Figure 11E, the levels of immune cell infiltration in the high-risk group were generally lower than those in the low-risk group, especially for CD8+T cells, neutrophils, natural killer (NK) cells, T helper (Th) cells (Tfh, Th1, and Th2 cells), tumor-infiltrating lymphocytes (TILs) and regulatory T (Treg) cells. Moreover, the immune-related pathways were less active in the high-risk group than in the low-risk group (Figure 10D). To further confirm the correlation of lncRNA signatures with the immune microenvironment, we investigated the abundance of 22 different immune cell types between the low- and high-risk groups using CIBERSORT algorithm (Figure 10E). We found that half of the 22 immune cell types (B cells memory, CD4 memory-activated T cells, CD8 memory-activated T cells, dendritic cells, macrophages, monocytes, neutrophils, regulatory T cells, follicular helper T cells, T17 helper and T2 helper cells) were significantly differentially expressed in the two risk groups, and all of them were downregulated in the high-risk group compared with the low-risk group (Figure 10F). Furthermore, the risk score was negatively correlated with the abundance of eosinophil cells, CD8 memory-activated T cells, CD4 memory-activated T cells, B memory cells and regulatory T cells (Tregs) (Figure 10G). Also, we assessed the potential relationship between lncRNA signatures expression and the abundance of immune cells, and observed that most immune cells were significantly associated with the lncRNA signatures (Supplementary Figure S3E). These results indicated that the m6A-related lncRNAs were involved in the immune cell infiltration of CRC.

### Identification and validation of immune genes shared by lncRNA signatures

**FIGURE 12:**
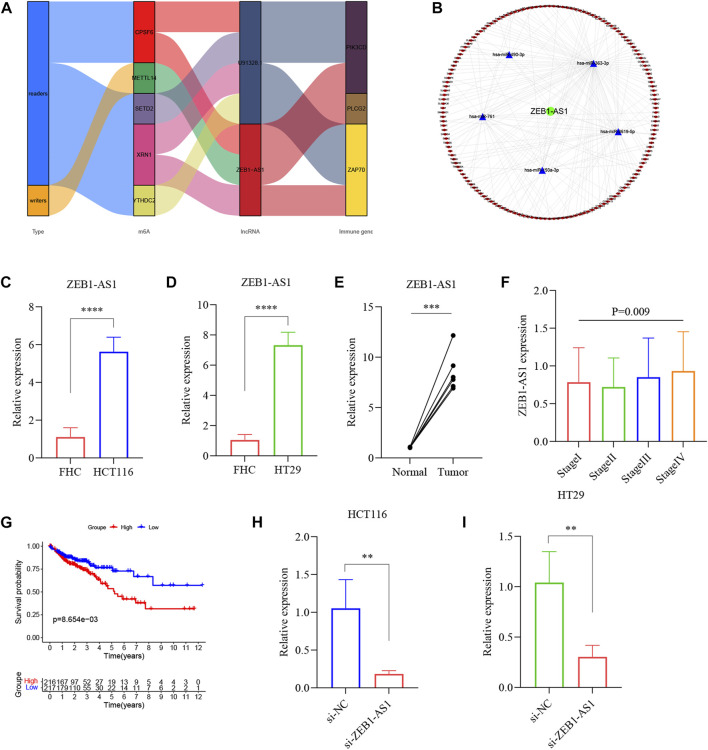
Validation of ZEB1-AS1 in CRC tissues and cell lines. **(A)** The sankey diagram depicted the relationship between 5 m6A regulators, 2 lncRNA signatures and 3 immune genes. **(B)** The ceRNA network based on 2 lncRNA signatures. **(C,D)** Bar plot for the relative expression of ZEB1-AS1 in CRC cell lines and normal cell line. **(E)** Bar plot for the relative expression of ZEB1-AS1 between CRC tissues and normal tissues. **(F)** The correlation of ZEB1-AS1 expression with clinicopathological staging characteristics. **(G)** Survival analysis for CRC patients with different ZEB1-AS1 expression. Patients were labeled with high expression or low expression depending on the comparison with the median expression level. **(H,I)** Transfection efficiency of ZEB1-AS1 siRNA was determined by RT-qPCR. **p < 0.01; ***p < 0.001; ****p < 0.0001.

Given the differential of the ImmuneScore between the high- and low-risk group, we further attempted to perform the Kaplan-Meier survival analysis. As shown in Figure 11A, the OS in the high ImmuneScore group was longer than that in the low ImmuneScore group. To ascertain the exact changes in the genetic profiles in the TME regarding immune components, variance analysis of high and low scores was performed by setting FDR <0.05 and |log2 FC| ≥ 1. A total of 2764 differentially expressed immune genes (DEIGs) were obtained from ImmuneScore with the cut-off value 56.042 of 441 CRC patients (Table S2). Further, 458 DEIGs having the 8 lncRNA signatures were screened using the correlation analysis (|Pearson R| > 0.1 and FDR <0.05) (Figure 11B). Among them, 456 genes were up-regulated and 2 genes were down-regulated (Figure 11C, Supplementary Table S3). To further explore the underlying mechanism, a protein–protein interaction (PPI) network was conducted based on STRING (version 11.5) with the highest confidence score of 0.9. The interactions between 458 genes are shown in Figure 11D, and the cytoscape plug-in cytoHubba was utilized to determine the top 50 hub immune genes (Figure 11E). Univariate Cox regression analysis for the survival of CRC patients was performed to determine the significant factors among 458 DEIGs (Figure 11F). Further, the common immune genes were identified from two lists, which were top 50 hub immune genes in PPI network and significant prognosis factors in univariate Cox regression. Finally, only 3 immune genes, named PIK3CD, PLCG2 and ZAP70, were overlapping from the above analyses (Figure 11G). Wilcoxon rank sum test revealed that the expression of the 3 immune genes in the tumor samples were all significantly lower than that in the normal samples (Figure 11H), which was consistent with the result of GEPIA 2 database (Supplementary Figure S6A). In addition, the survival analysis showed that CRC patients with 3 immune genes low expression had longer survival than that high expression (Figure 11I). For further validation, we used TISCH to depict the expression distribution of 3 immune genes in the TME of CRC. It was found that PIK3CD was enriched in NK cells, CD8 + T cells, CD8 + Tex, CD4 + Tconv cells, mononuclear/macrophage subsets and B cells. PLCG2 was abundant in NK cells, CD8 + T cells, CD8 + Tex, mononuclear/macrophage subsets, B cells and plasma. ZAP70 was essentially distributed in NK cells, CD8 + T cells, CD8 + Tex, CD4 + Tconv cells and T proilf cells (Figure 11J). These results suggest that these immune genes may be the downstream regulators of m6A regulators and related lncRNAs participated in TME remodeling (Figure 12A).

### The correlation of lncRNA ZEB1-AS1 expression with epithelial/mesenchymal transition and chemoresistance in colorectal cancer patients

**FIGURE 13:**
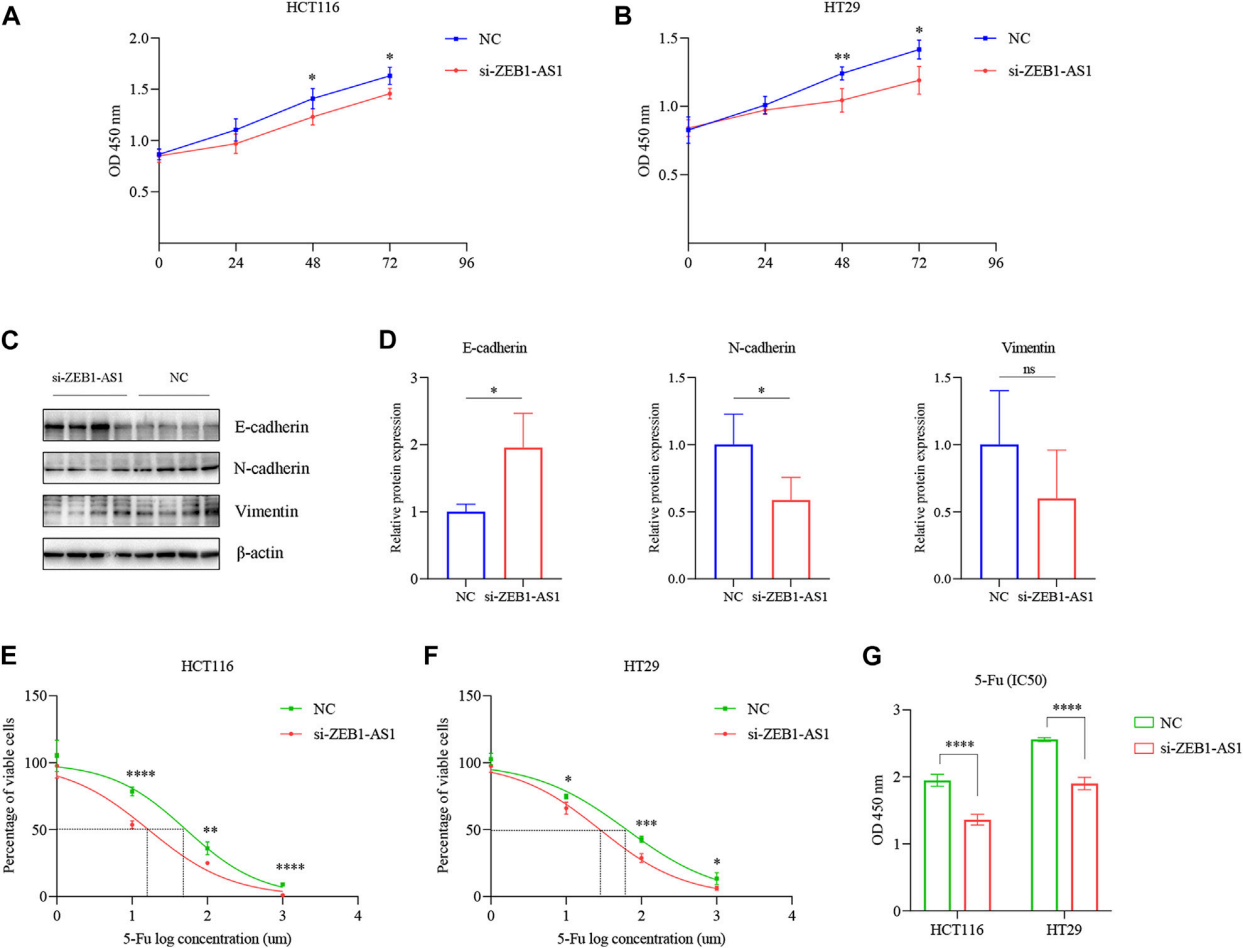
ZEB1-AS1 promotes the CRC cell proliferation, metastasis of EMT and chemoresistance to 5-Fu *in vitro*. **(A,B)** The proliferative ability of HCT116 and HT29 cells was determined by CCK8 assay. **(C,D)** Protein levels of E-cadherin, N-cadherin, and Vimentin were measured by Western blot. Data are presented as means ± SD. **(E,F)** Cells were transfected by ZEB1-AS1 siRNA and then exposed to serial dilutions of 5-Fu for 48 h (cell viability was determined by CCK8 assay and data are presented as the percentage of viable cells). **(G)** Cells were transfected by ZEB1-AS1 siRNA and then in the presence of IC50 5-Fu for 48 h (cell viability was determined by CCK8 assay and data are presented as the percentage of viable cells). *p < 0.05; **p < 0.01; ***p < 0.001; ****p < 0.0001.

Increasing evidence indicates that ceRNAs regulatory networks serve essential roles in the occurrence, development, and regulation of tumors. Thus, we put the ZEB1-AS1 and U91328.1 (related to immune genes) into the miRcode11 database (highly conserved miRNA families) to identify potential miRNA-targeting lncRNAs. To enhance the accuracy of the prediction, we then identified the downstream target mRNAs that were shared by only three databases: miRDB, miRTarBase, and TargetScan. Finally, cytoscape was used to construct a regulatory network of ceRNAs, including 1 m6A-related lncRNA (ZEB1-AS1), 5 miRNAs, and 152 mRNAs (Figure 12B). Additionally, multivariate analyses revealed that ZEB1-AS1 expression was independent risk factors affecting the prognosis of CRC patients (Supplementary Figure S6B). Further, the expression of ZEB1-AS1 was verified in 6 pairs of CRC tissues and normal colon tissues and human colon mucosal epithelial cell line FHC and human CRC cell lines HT29 and HCT116 by RT-qPCR. As shown in Figures 12C–E, the expression of ZEB1-AS1 was significantly upregulated in CRC cell lines and tumor tissues, consistent with the findings of our studies. In addition, our results revealed that the expression of ZEB1-AS1 was related to the pathologic stage (*p* = 0.009), and high expression of ZEB1-AS1 was also associated with poor prognosis in CRC patients based on the median of ZEB1-AS1 expression levels (*p* = 0.0086) (Figures 12F,G). In the GEPIA 2 database, the results of ZEB1-AS1 were consistent with ours, suggested that ZEB1-AS1 was involved in the malignant progression of CRC (Supplementary Figures S6C–S6H). Also, the TISCH database was used to analyze the relationship between ZEB1-AS1 expression and staging in different subsets of cells. As shown in Supplementary Figure S6I, ZEB1-AS1 was significantly correlated with staging in the mononuclear/macrophage subgroup. To further explore the biological function of ZEB1-AS1 on CRC, we analyzed the correlation ZEB1-AS1 expression with the scores of the associated signaling pathways. As is shown in Supplementary Figures S7A–S7S, most of the signaling pathways in the CRC, especially ZEB1-AS1 showed a high level of activation in the TGF-β, EMT, angiogenesis, collagen formation and degradation of extracellular matrix signaling pathway, but consistent inhibition of the cellular response to hypoxia, DNA damage response, MYC target response and accumulation of reactive oxygen species (ROS) signaling pathways. Epithelial/Mesenchymal Transition (EMT) is one of the core mechanisms of tumor metastasis, and it is also one of the main factors leading to poor prognosis of patients. Interestingly, correlation analysis showed that ZEB1-AS1 was significantly positive correlated with N-cadherin and vimentin (Supplementary Figure S7T). For further validation, ZEB1-AS1 siRNA was transfected into HT29 and HCT116 cells (Figures 12H,I). Reduction in ZEB1-AS1 significantly inhibited the proliferation ability of HT29 and HCT116 cells (Figures 13A,B). Through the western blot analysis, we found that EMT marker E-cadherin was up-regulated, while N-cadherin was down-regulated in ZEB1-AS1 siRNA transfected CRC cells (Figures 13C,D). Notably, the protein level of Vimentin had no significant correlation with the ZEB1-AS1 expression. In addition, 5-fluorouracil (5-Fu) chemoresistance is a major challenge and the prognosis for CRC patients can be very poor due to recurrence of disease (Brenner et al., 2014). We further focused on the correlation of ZEB1-AS1 expression with the sensitivities of 5-Fu (IC50 value) based on Genomics of Drug Sensitivity in Cancer (GDSC). Interestingly, the IC50 values of 5-Fu suggested a positive association with the expression levels of ZEB1-AS1 (*p* = 0.021) (Supplementary Figure S6J). Unsurprisingly, our experiments results suggested that ZEB1-AS1 siRNA increased cytotoxicity induced by 5-Fu in HCT116 and HT29 cells (Figures 13E,F). In addition, the IC50 value of 5-Fu in HCT116 and HT29 cells was approximately 50 μM and 60 μM, and ZEB1-AS1 siRNA increased IC50 value of 5-FU induced apoptosis in HCT116 cells and HT29 cells compared with controls (Figure 13G). Therefore, these results indicated that elevated ZEB1-AS1 levels can promote carcinogenesis, metastasis of EMT and the chemoresistance of 5-Fu in CRC.

## Discussion

After witnessing the success of targeted molecular therapy in some tumor clinical applications, there has been increasing enthusiasm for research on the impact of lncRNAs on CRC (Chang et al., 2021; Zhang et al., 2021d). In addition, an increasing number of studies have shown that the heterogeneity of CRC is characterized by distinct clinical features, prognosis and therapy response (Linnekamp et al., 2015; Zhai et al., 2017), and the differing expression of noncoding RNAs has been revealed to be associated with distinct clinical features, therapy response and clinical outcomes (Zhou et al., 2018). However, the specific influencing mechanism of m6A modification on prognostic lncRNAs remains rare. Moreover, the overall mechanism of lncRNAs in CRC is not yet completely understood.

In this study, 1707 m6A-related lncRNAs, using the TCGA dataset, were selected in order to explore their association with the TME, progression and prognosis of CRC. First, 28 m6A-related lncRNAs were confirmed to be associated with the OS in CRC patients. Subsequently, two different subtypes were identified based on the 28 m6A-related lncRNAs, and cluster 2 appeared to have poor immune cell infiltration and clinical results. Further, 8 of the m6A-related lncRNAs were successfully used to construct an m6A-related lncRNA signatures model. According to the intermediate risk score, CRC patients were divided into high- and low-risk groups, and our results showed that significantly different clinicopathological characteristics, prognosis, mutation, TME, immune checkpoints, MSI, CSC index, and drug susceptibility between the two risk groups. In addition, multivariate Cox regression analysis indicated that the lncRNA signatures could act as an independent predictor, and the AUC values obtained confirmed the signatures’ high predictive power for CRC patients. We also constructed a nomogram showing that the 1-, 3-, and 5- year OS rates could be predicted relatively well. Further, we screened 3 downstream immune genes targeted by the lncRNA signatures based on the hub and prognostic analysis. Recently, PIK3CD has been reported to be associated with the homeostasis and function of B and T cells, and can induce cancer cell growth and invasion by activating AKT/GSK-3β/β-catenin signaling in CRC (Chang et al., 2021; Zhang et al., 2021d). PLCG2 is determined as a potential indicator of TME remodeling in tumor, and dysfunction of PLCG2 is closely associated with inflammation, immune disorders, and cancer (Koss et al., 2014; Li et al., 2021b). ZAP70 has been found to be a master regulator of adaptive immunity, and is essential for long-term survival of naive CD8 + T cells (Fischer et al., 2010; Schim van der Loeff et al., 2014). Subsequently, a Sankey diagram that depicted the relationship between m6A methylation regulators, lncRNAs, and immune genes was constructed. These results suggest that lncRNA signatures may affect the immune cell infiltration through 3 common immune genes and provide invaluable insights into the future treatment of patients with CRC. Finally, we determined that ZEB1-AS1 was the most significant lncRNA signatures in CRC, and through *in vitro* experiments, we were able to verify our conclusions. ZEB1-AS1 has been reported that promotes PAK2 expression by sponging miR-455-3p and further facilitating CRC cell growth and metastasis (Ni et al., 2020). In addition, a recent study revealed that ZEB1-AS1 is an independent prognostic factor for patients with advanced gastric cancer receiving chemotherapy (Chai et al., 2019). ZEB1-AS1 promotes paclitaxel and cisplatin resistance by regulating MMP19 in ovarian cancer (Dai et al., 2021). ZEB1-AS1 promotes triple-negative breast cancer resistance to doxorubicin through miR-186-5p/ABCC1 signaling (Lu et al., 2021). However, current studies have focused on the effect of ZEB1-AS1 on cancer proliferation, invasion and metastasis, but little information is available regarding the effects of ZEB1-AS1 on chemoresistance. Here, we found that ZEB1-AS1 was overexpressed in CRC and conferred independent risk factors affecting the prognosis to CRC patients. Further *in vitro* studies were performed to verify that ZEB1-AS1 was involved in 5-Fu resistance in CRC. In summary, m6A-related lncRNAs play a critical role in malignant progression of CRC. Understanding the potential molecular mechanism of these lncRNAs in CRC is important to improve the knowledge of molecular biological basis of CRC development and identify novel special biomarkers or therapeutic target for CRC patients.

Immune cells, such as granulocytes, lymphocytes, and macrophages are major cellular components of TME. Increasing evidence has shown that effector T cells, memory T cells, and T cell differentiation play a vital role in the immune defense of CRC (Chang et al., 2021; Zhang et al., 2021d). Increasing efforts in colon cancer immunogenomics have shown that CRC tissues with higher densities of tumor-infiltrating T cells indicate a good prognosis (Ma et al., 2020). In our study, cluster 1 and low risk score, with a better prognosis, showed higher infiltration of activated memory CD4 + and CD8 + T cells, suggesting that they play a positive role in CRC development. Further, our lncRNA signatures showed that the low-risk group was closely associated with a high ImmuneScore. We revealed that the levels of antitumour immune infiltrations and the activity of immune-related pathways in the high-risk group were lower than those in the low-risk group, which indicated that the immune functions of the high-risk group were overall impaired. Thus, the difference in survival rates between the low- and high-risk groups might have been caused by differing immune infiltration. The infiltration of Tregs, which suppress the anti-cancer immune response, was associated with poor prognosis (Tanaka and Sakaguchi, 2017). Astonishingly, our study found that the number of Treg cells in the low-risk group was significantly higher than that in the high-risk group (*p* < 0.01). One reason for this inconsistency may be that two main opposite roles of the subtypes of Treg cells in the TME of CRC exist (Saito et al., 2016). Therefore, it is crucial for future studies to identify the subtypes of Treg cells in CRC. Recent studies revealed that B cells also participate in the immune response, and was positively correlated with the response to PD-1 blockade in soft-tissue sarcomas (Helmink et al., 2020; Petitprez et al., 2020). In addition, tumor-infiltrating B cells were associated with a favorable prognosis in CRC (Chang et al., 2021; Zhang et al., 2021d). The high B cell infiltration have a significantly lower risk of disease recurrence and prolonged overall survival in metastatic CRC patients (Meshcheryakova et al., 2014). In our study, we observed the abundance of memory B cells in cluster B and high-risk groups with worse overall survival were significantly lower than those in cluster A and low-risk groups, suggested that patients with cluster A and low-risk groups might benefit from immunotherapy. Thus, infiltration of B cell inhibited tumor progression in CRC, consistent with the findings of previous studies. Eosinophils have been shown to infiltrate multiple tumors, either as an integral part of the TME or in response to various therapeutic strategies (Grisaru-Tal et al., 2020). Furthermore, high tumor stromal eosinophil score has been associated with a decreased risk for all-cause and colorectal cancer death (Fernandez-Acenero et al., 2000; Prizment et al., 2016). Consistent with previous publications, we noticed an increased infiltration of eosinophils in cluster 1 and low risk score groups with a favorable prognosis, which suggesting that eosinophils play a role in the TME and can affect the prognosis and response to therapy in CRC. In future, single-cell RNA sequencing should be exploited to help dissect the diverse phenotypic landscape of CRC infiltrating eosinophils under physiological conditions and following therapy. Overall, our study revealed that the m6A-related lncRNAs play a vital role in TME infiltration, and it provides a new insight into the association of immune infiltration with clinical significance in CRC patients.

Nonetheless, our study has certain limitations. First, due to the lack of available data about lncRNA sequencing in the Gene Expression Omnibus (GEO) database and other databases, further verification could not be performed. We will continue to follow up sequencing cases in the future to improve the prediction model based on the data from our center. Several studies have adopted |Pearson’s R| > 0.3 and *p* < 0.001 or |Pearson’s R| > 0.5 and *p* < 0.001 to screen m6A-related lncRNAs(Zhang et al., 2021b; Zhang et al., 2021e). However, whether Pearson’s R| > 0.3 or 0.5 could not classify the patients into distinct molecular subtypes well in our study (*p* > 0.05, data not shown). Thus, our process used the criteria of |Pearson’s R| >0.4 and *p* < 0.001 to identify the m6A-associated lncRNA, which was consistent with previous study (Liu et al., 2021). Whether the other |Pearson’s R| values can affect the results of the study deserve further investigation. Additionally, most of the results were predicted by bioinformatics analysis, further experiments *in vitro* or *in vivo* are needed to demonstrate the associations between these factors.

## Conclusion

In this study, our results provide a novel lncRNA signatures model for predicting the prognosis of CRC patients. Moreover, the lncRNA signatures between the low- and high-risk group were closely associated with the immune infiltration, and this offers a significant basis for future studies on the relationships between m6A-related lncRNA and TME in CRC. We also determined the therapeutic liability of lncRNA signatures in targeted therapy, immunotherapy, and chemotherapy. These findings highlight the crucial clinical implications of m6A-related lncRNA and provide new ideas for guiding personalized therapy strategies in CRC patients. In addition, high expression of ZEB1-AS1 can be used as a molecular marker to identify CRC patients in high-risk groups, and a potential therapeutic target to improve the survival of CRC patients.

## Data Availability

The original contributions presented in the study are included in the article/Supplementary Materials, further inquiries can be directed to the corresponding authors.

## Supplementary material

SUPPLEMENTARY FIGURE S1 The differential expression levels of 28 m6A-related prognostic lncRNAs in normal and tumor tissues. **p* < 0.05; ***p*< 0.01; ****p* < 0.001.

SUPPLEMENTARY FIGURE S2 The Gene Set Enrichment Analysis (GSEA) between two clusters based on 28 m6A-related prognostic lncRNAs.

SUPPLEMENTARY FIGURE S3 ROC curves of the clinical characteristics and risk score. **(B–D**) The Kaplan-Meier survival curves of the relative DFI, PFI, and DSS between the high- and low-risk group. **(E)** Correlations between the abundance of immune cells and 8 m6A-related lncRNA signatures in the proposed model. **(F)** Expression of immune checkpoints in the high and low-risk groups. **p* < 0.05; ***p*< 0.01; ****p* < 0.001.

SUPPLEMENTARY FIGURE S4 The Kaplan-Meier survival curves of OS differences stratified by age, gender, pathologic stage, and TMN stages between the high- and low-risk group.

SUPPLEMENTARY FIGURE S5 Relationships between lncRNA signatures and drug-sensitivity (IC50 value).

SUPPLEMENTARY FIGURE S6 Box plots of individual immune genes expression in CRC based on GEPIA analysis. **(B)** The multivariate cox forest plot of the immune genes related two lncRNA signatures in CRC by R package “survival” and “forestplot.” **(C)** Verification of the expression level of ZEB1-AS1 in GEPIA database. **(D,I)** Correlation between ZEB1-AS1 expression and tumor stage of CRC in GEPIA and TISCH database. **(E–H)** The prognostic value of mRNA level of ZEB1-AS1 in CRC (Kaplan–Meier plotter) based on the median and quartile distribution of ZEB1-AS1 expression levels. **(J)** Correlation heatmap of different expressed ZEB1-AS1 with EMT marker genes. **p* < 0.05.

SUPPLEMENTARY FIGURE S7
**(A–S)** The relationships between the expression of ZEB1-AS1 and important cancer signaling pathways in CRC. **(T)** The correlation between the expression of ZEB1-AS1 and the drug sensitivity of 5-Fu.

SUPPLEMENTARY TABLE S1 The primer sequences for qPCR.

SUPPLEMENTARY TABLE S2 The 2764 differentially expressed immune genes between high- and low-ImmuneScore groups.

SUPPLEMENTARY TABLE S3 The correlations between lncRNA signatures and 458 differentially expressed immune genes.
