# Supplementary figures and images for: Identification of N6-methylandenosine related lncRNA signatures for predicting the prognosis and therapy response in colorectal cancer patients
Source: Front Genet. 2022 Sep 30;13:947747. doi: 10.3389/fgene.2022.947747 (PMC9561883; doi:10.3389/fgene.2022.947747)

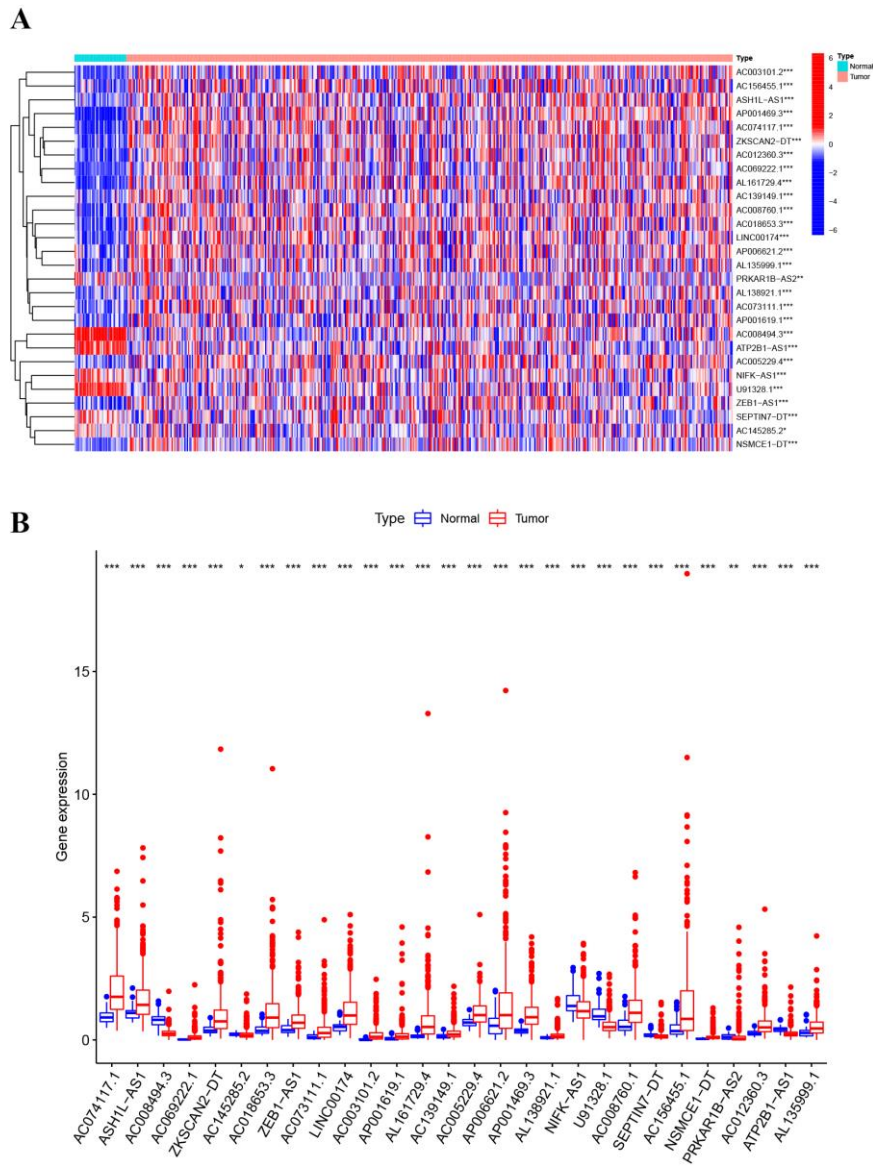

FIGURE S1

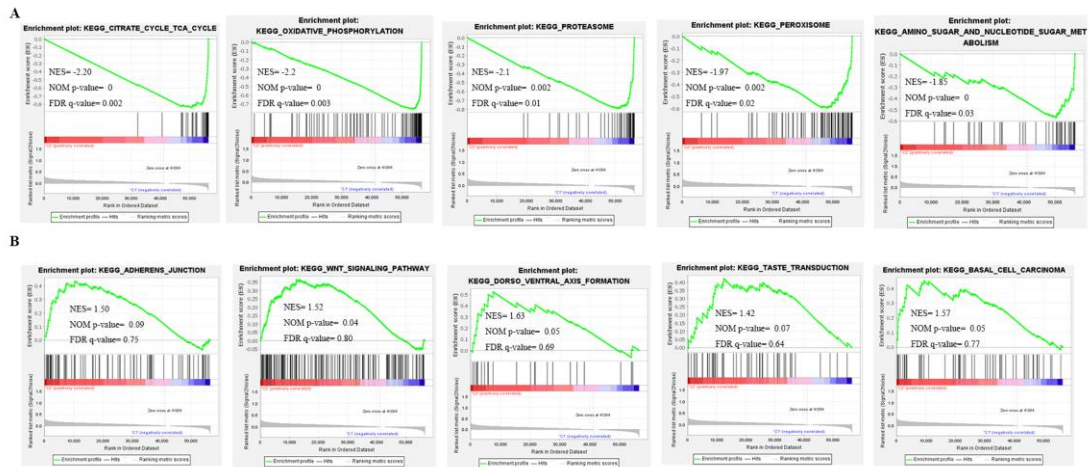

FIGURE S2

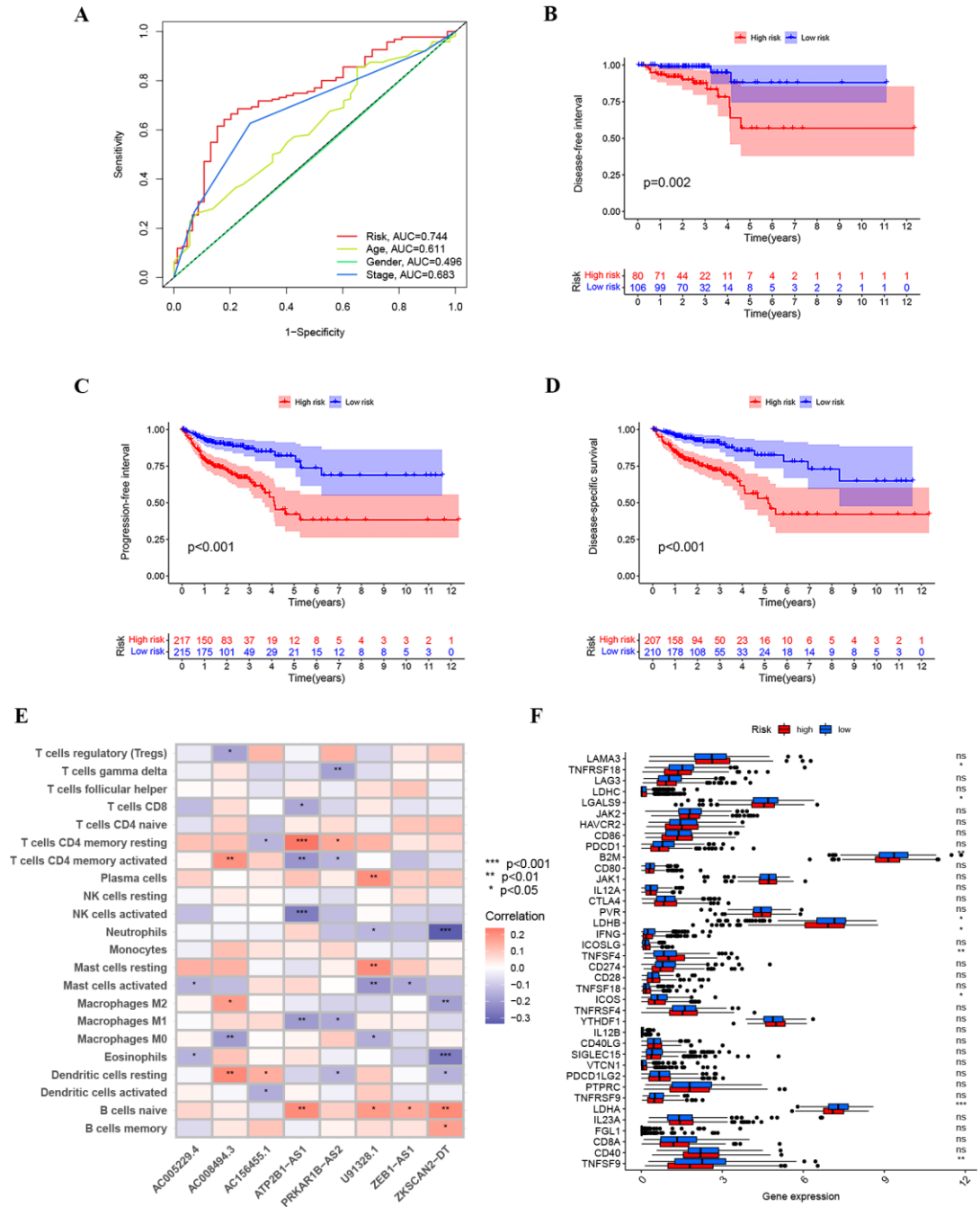

FIGURE S3

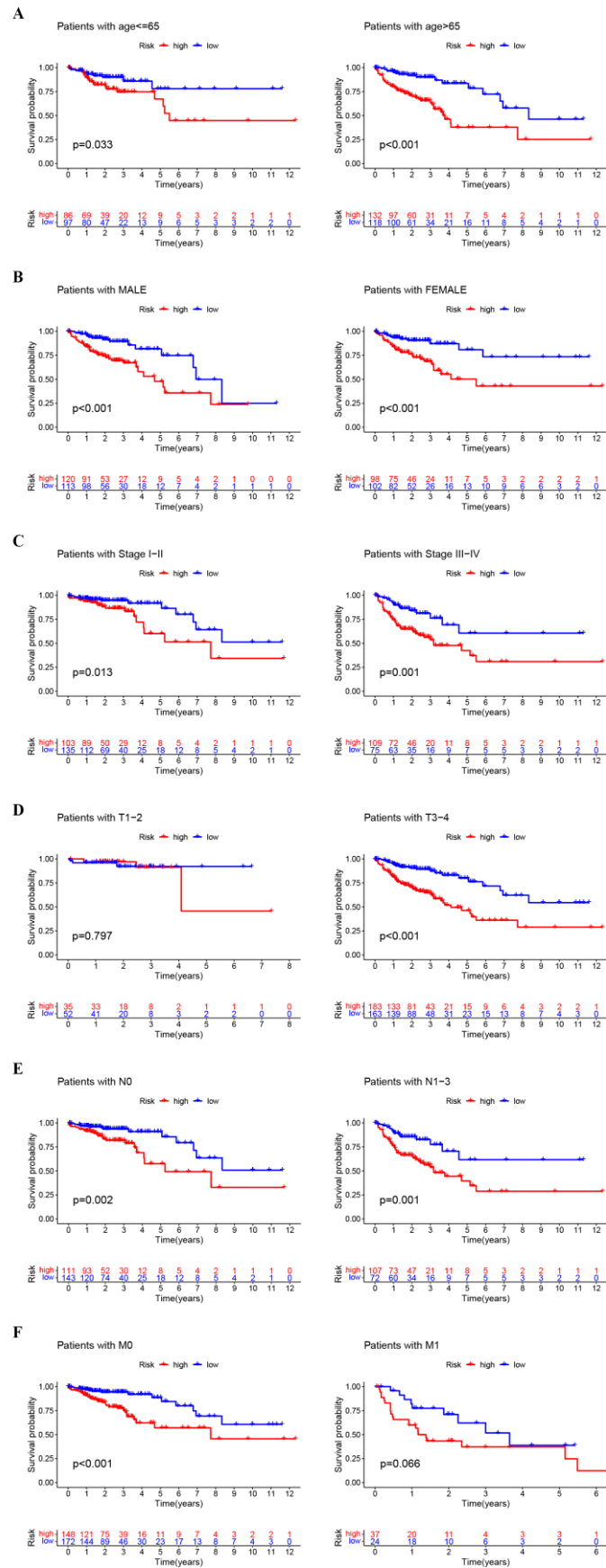

FIGURE S4

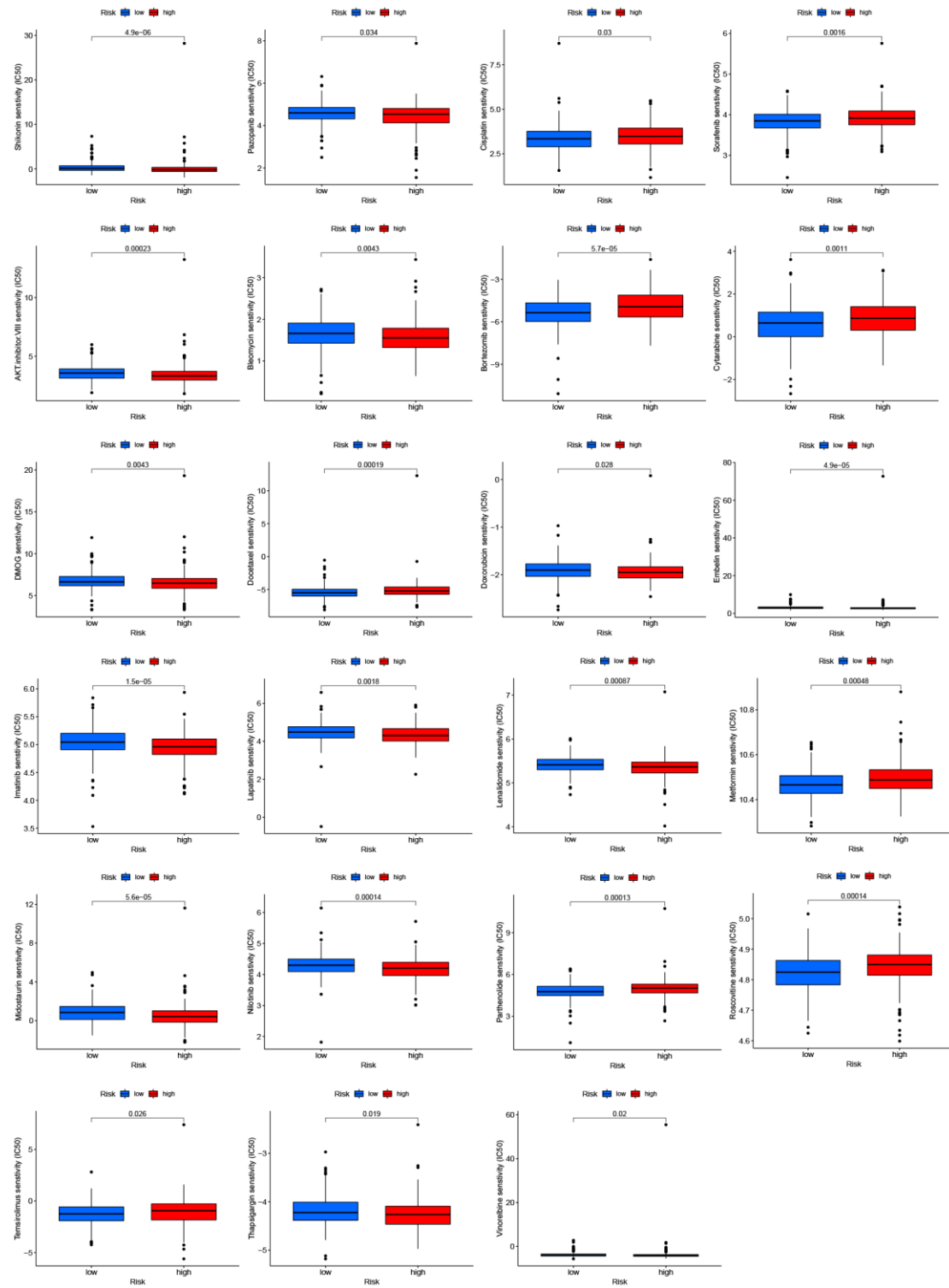

FIGURE S5

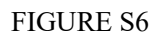

FIGURE S6

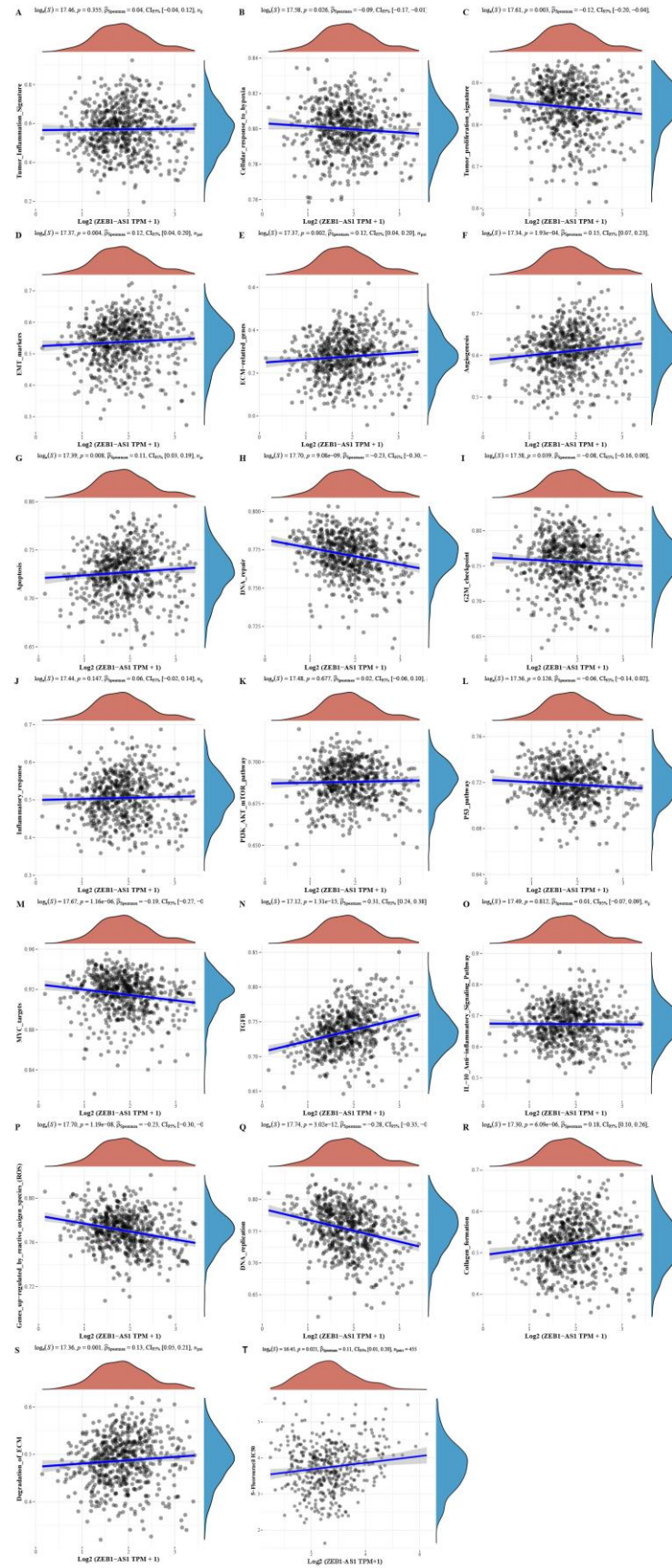

FIGURE S7

Supplement: Supplementary file 3 [file DataSheet1.PDF]
